# Paediatric brainstem: A comprehensive review of pathologies on MR imaging

**DOI:** 10.1007/s13244-016-0496-3

**Published:** 2016-05-23

**Authors:** Chandan Kakkar, Shruti Kakkar, Kavita Saggar, Jatinder S. Goraya, Archana Ahluwalia, Ankur Arora

**Affiliations:** Department of Radiodiagnosis and Imaging, Dayanand Medical College and Hospital, Ludhiana, India; Division of Pediatric Haemato-oncology, Department of Pediatrics, Dayanand Medical College and Hospital, Ludhiana, India; Division of Pediatric Neurology, Department of Pediatrics, Dayanand Medical College and Hospital, Ludhiana, India; Worthing Hospital, Western Sussex NHS Foundation Trust, Lyndhurst Road, Worthing, BN112DH UK

**Keywords:** Brainstem, MRI, Demyelination, Encephalitis, Glioma

## Abstract

The brainstem is a midline structure formed by the midbrain, pons and medulla and is a home for various vital neurological centres of the human body. A diverse spectrum of disease entities can involve the brainstem, which includes infections, metabolic disorders, demyelination, vascular conditions, neurodegenerative disorders and tumours. Brainstem involvement can be primary or secondary, i.e., as part of systemic disorders. Due to the overlapping clinical presentation and symptomatology, imaging plays a decisive role in the detection, localisation and characterisation of brainstem pathologies. Magnetic resonance imaging (MRI) is the modality of choice and the use of advanced MR techniques such as diffusion-weighted imaging and spectroscopy can be especially helpful in providing a tenable diagnoses. This article is a compilation of the MR imaging manifestations of a spectrum of common and uncommon brainstem pathologies that can be encountered in the paediatric age group.

*Teaching Points*

*• The paediatric brainstem can be afflicted by many pathologies that may overlap clinico-radiologically.*

*• MRI is the best modality for the localisation and diagnosis of brainstem pathologies.*

*• Diffusion-weighted imaging is useful in the diagnosis of vascular and metabolic disorders.*

*• Occasionally, demyelination and neoplasms can be indistinguishable on imaging.*

## Introduction

Pathologies involving the paediatric brainstem have always been a diagnostic dilemma for paediatric neurologists as well as radiologists. Typically, the presence of long tract signs and cranial nerve palsies favour brainstem involvement; however, brainstem pathologies can have a varied presentation. Long-standing disorders can manifest with Parinaud syndrome, which suggests involvement of the dorsal midbrain, whilst the presence of internuclear ophthalmoplegia connotes involvement of the dorsomedial pons or tegmentum. Diagnosing brainstem pathologies solely on clinical considerations is not always feasible, for example, patients with acute vascular events or encephalitis typically present with altered sensorium or in a comatose state which make brainstem localisation virtually impossible. Imaging thus comes to play a paramount role in the detection and localisation of the abnormalities to the brainstem. It is however vital to understand that on imaging there can be a considerable overlap of radiological findings, with differential diagnoses ranging from infections, demyelination, to high-grade brainstem gliomas. The histopathological diagnosis may not always be possible given the deep-seated nature of the lesions and associated morbidity that could result subsequently. Thus to achieve a plausible diagnosis imaging examinations need to be interpreted in the light of clinical details and laboratory parameters [1–3].

In this review, we present the clinico-radiological features of different brainstem pathologies in the paediatric population and discuss a comprehensive diagnostic approach to these myriad conditions. These disorders can be broadly classified into vascular pathologies, demyelination, metabolic, toxic and neurodegenerative, encephalitis and tumours (Table 1).

**Table 1:** Diseases of the paediatric brainstem

Diseases of the paediatric brainstem
Vascular:
Infarction
Haemorrhage
Hypoxia
Vasculitis
Vascular malformations
Demyelination:
ADEM
Multiple sclerosis
Neuromyelitis optica
Osmotic demyelination
Metabolic and neurodegenerative:
Leigh’s disease
Maple syrup urine disorder
Glutaric aciduria
Wilson’s disease
Encephalitis:
Viral
Tubercular
Fungal
Parasitic
Tumours:
Pontine glioma
Tectal glioma
Medullary glioma

## Vascular Disorders of the Brainstem

### Stroke

**Fig. 1:**
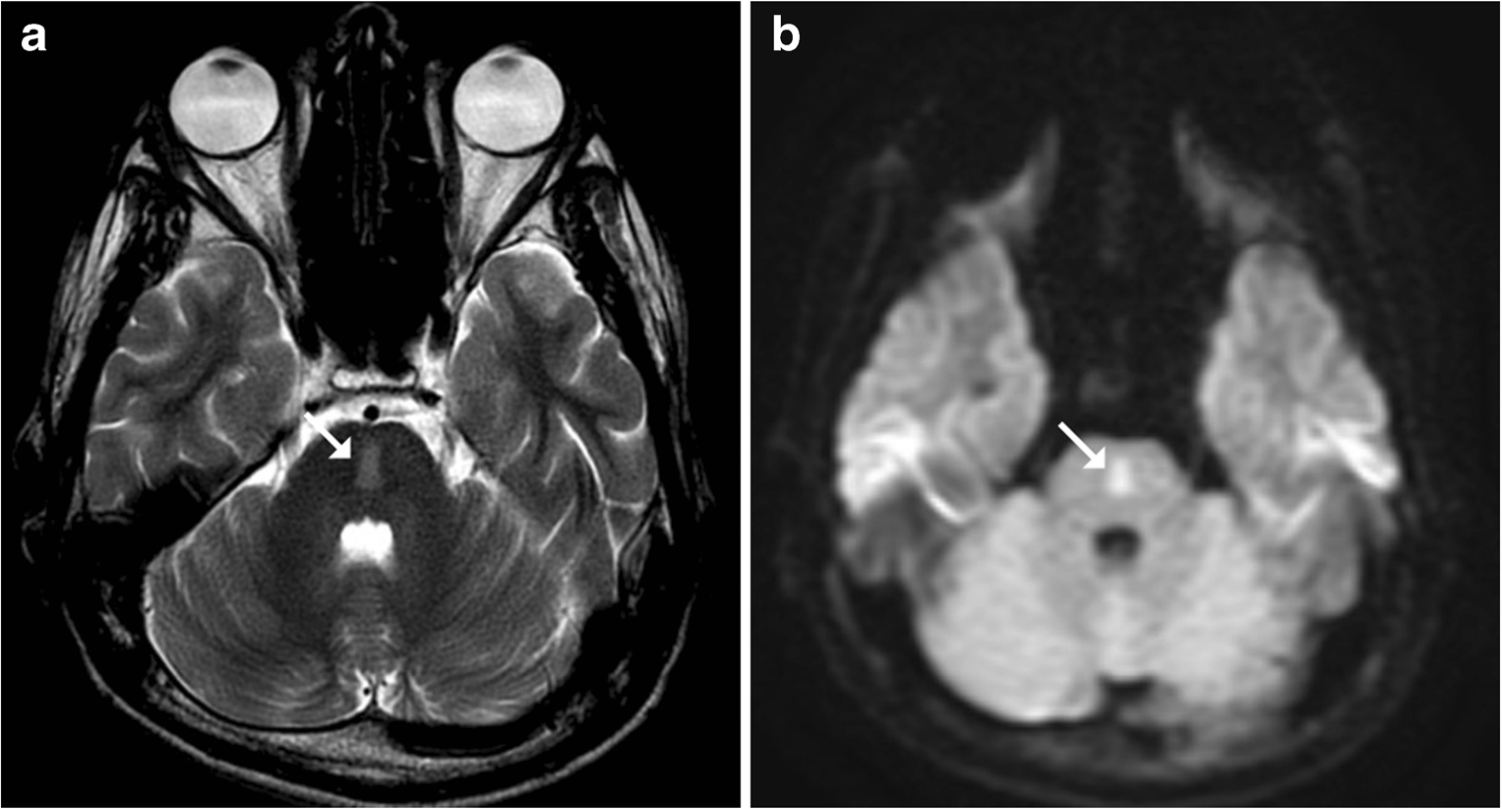
An 8-year-old patient with known acute leukaemia on induction therapy presented with bradycardia and hypotension: **a**–**b** Axial T2- and diffusion-weighted images reveal focal hyperintensity (*arrow*) showing diffusion restriction consistent with an acute infarct

Arterial ischaemic stroke remains the prime cause of cerebrovascular accidents in the paediatric age group. Some of the most important risk factors for paediatric stroke include cardioembolic phenomenon, haematological disorders such as sickle cell anaemia, infections, vasculitis, dissection and drug toxicity (L-asparaginase). Acute brainstem stroke in this age group typically presents with focal neurological deficits, hemiplegia, bulbar symptoms and rarely as locked-in syndrome. Thromboembolic infarcts usually manifest as a focal area of hyperintensity on T2-weighted and fluid attenuation inversion recovery (FLAIR) sequences in the involved region with diffusion restriction in acute stages (Fig. 1). Haemorrhagic strokes are generally sequelae of underlying vascular malformations or haematological disorders [4].

### Hypoxic Ischaemic Injury

**Fig. 2:**
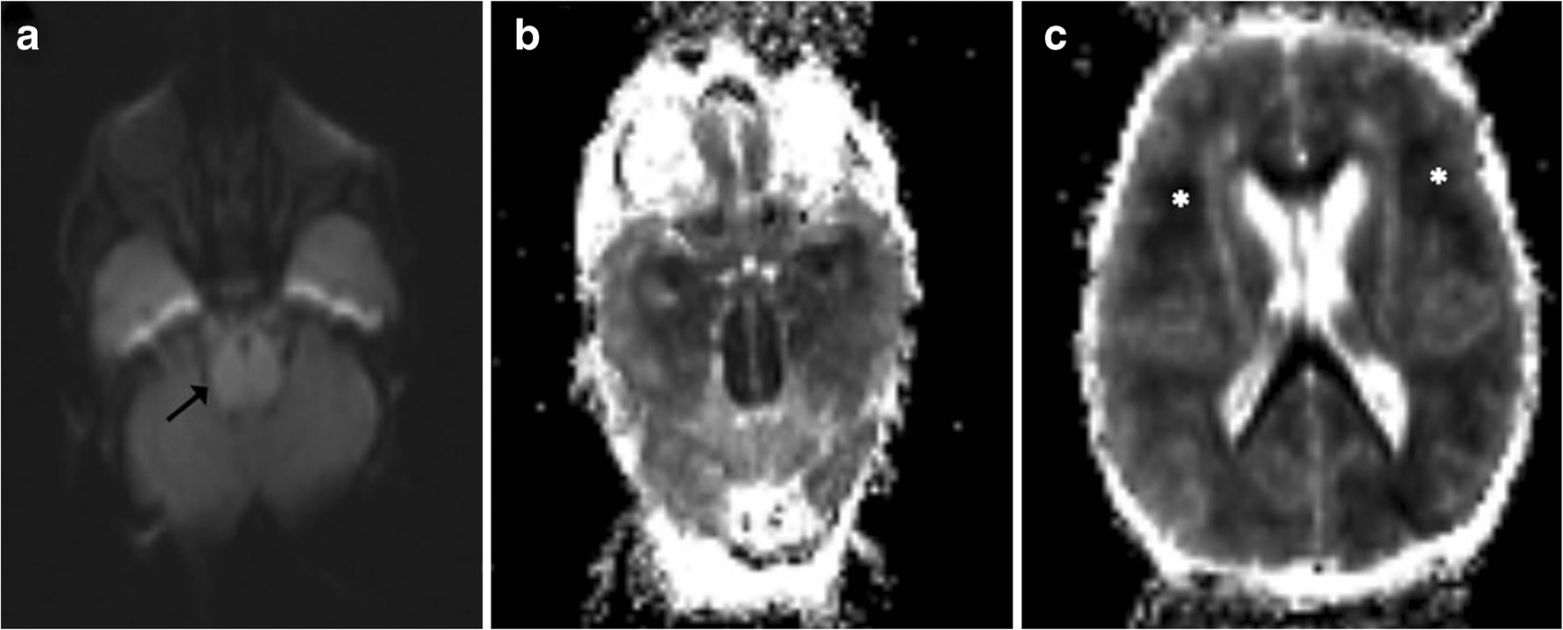
Neonate on the 5th day of life with a history of obstructed labour resulting in severe hypoxia: **a**–**b** Diffusion-weighted image shows hyperintense signal involving the pons (*arrow*) with corresponding signal changes on ADC. **c** Extensive diffusion restriction is also seen in the cerebral white matter (*asterisk*) consistent with severe hypoxia

**Fig. 3:**
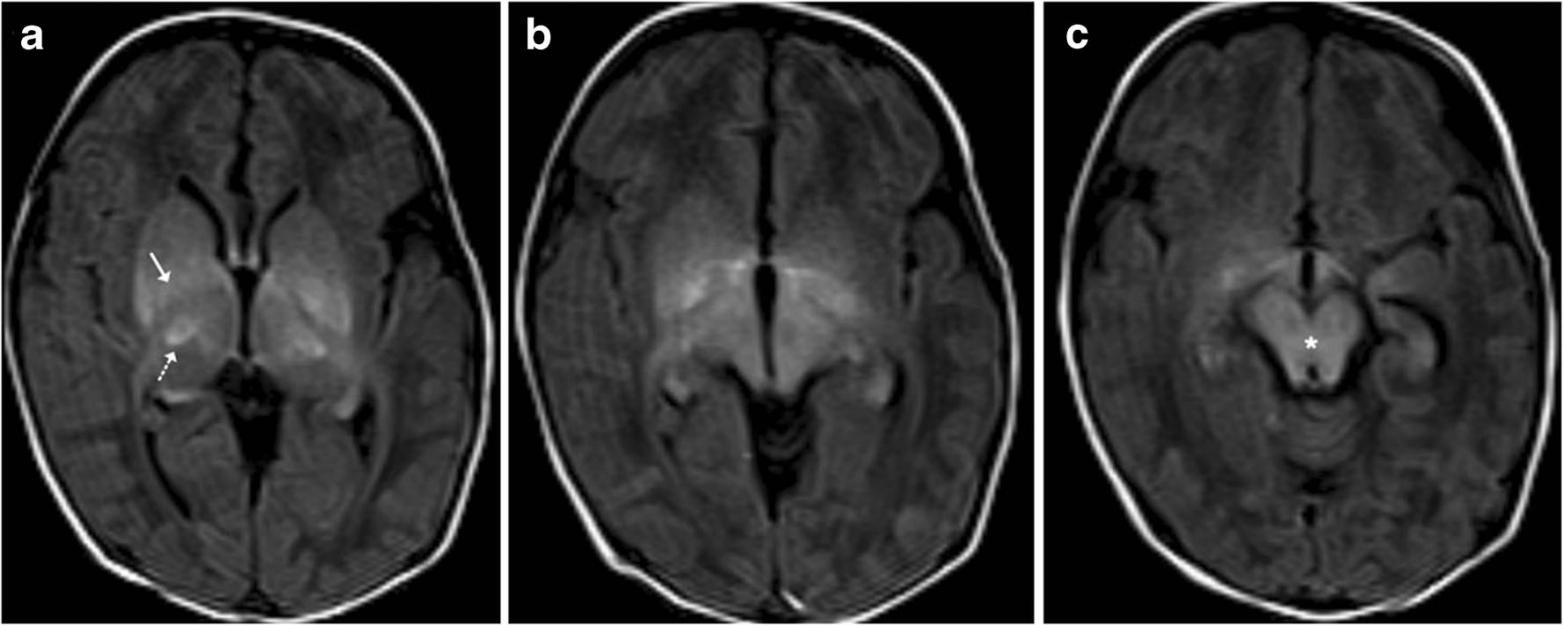
Neonate with a history of delayed cry: **a** T1-weighted image shows symmetric increased signal intensity involving the putamina (*arrow*) and ventrolateral thalami (*dotted arrow*). **b**–**c** Similar high signal intensity is also noted involving the midbrain (*asterisk*)

Profound hypoxia can involve the brainstem in the neonatal age group. Whilst the typical sites of involvement include the ventrolateral thalami and corticospinal tracts in the posterior limbs of the internal capsules, midbrain and the dorsal brainstem may also get involved. Diffusion-weighted imaging has the highest sensitivity in detecting early ischaemic necrosis, which manifests as areas of diffusion restriction (Fig. 2). After an initial progression the apparent diffusion coefficient (ADC) tends to normalise at 7–10 days. T1-weighted images can be especially helpful as they depict increased signal intensity within the ventrolateral thalami, posterolateral putamen, peri-Rolandic cortex and dorsal brainstem starting as early as the second day (Fig. 3) [5, 6].

#### Vasculitis (Behçet’s Disease)

**Fig. 4:**
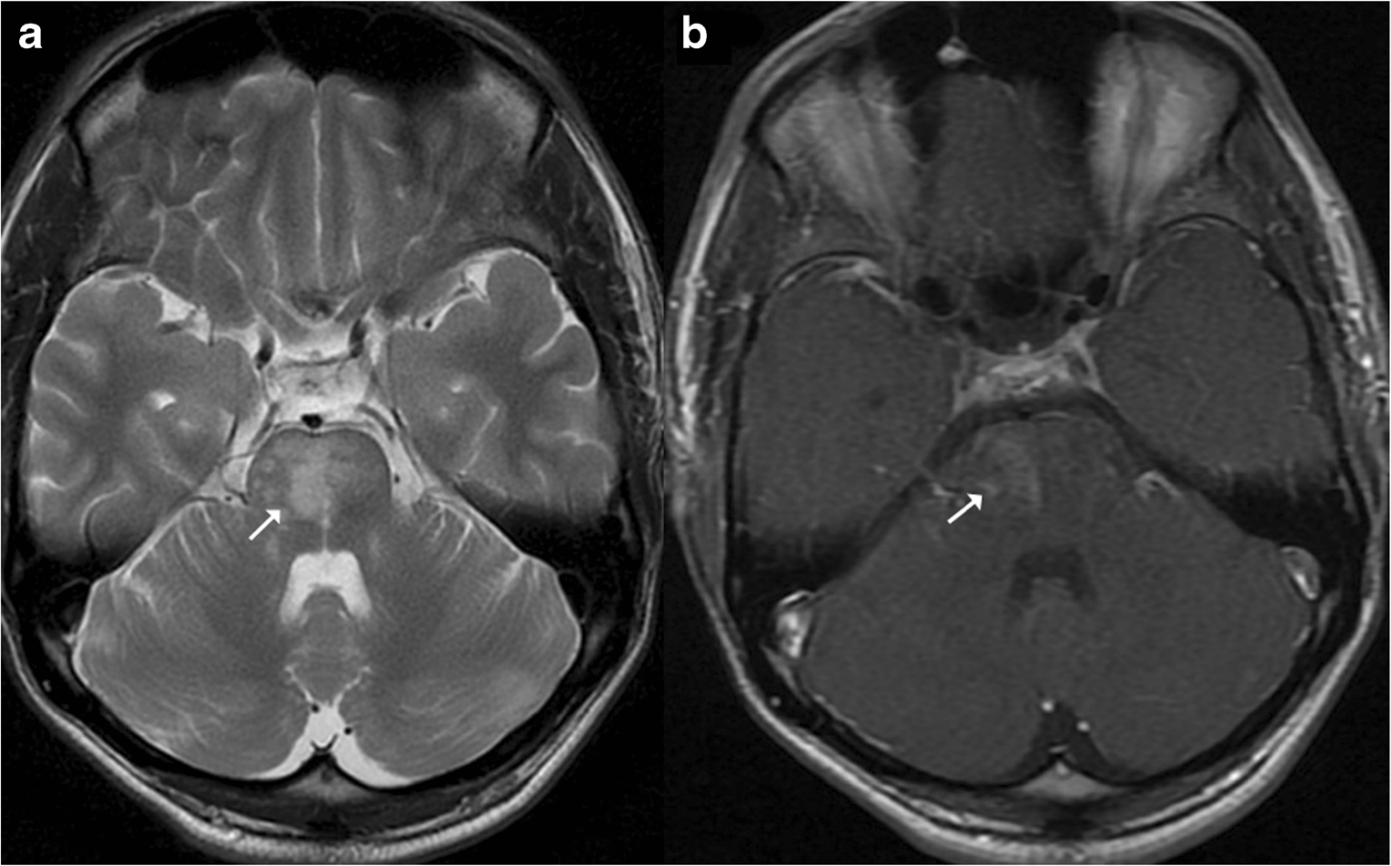
A 16-year-old patient known to have Behçet disease: **a** Axial T2-weighted image shows increased signal intensity in the pons (*arrow*). **b** Mild post-contrast enhancement is noted in the involved region (*arrow*)

Behçet’s disease is a multisystemic vasculitic disorder of unknown aetiology characterised by perivascular inflammatory reactions predominantly centred on the venules and arterioles. Paediatric age group disease has an onset in the 2nd decade with a mean age of neurological involvement being 13 years. The typical triad includes oral ulceration, genital ulceration and ocular manifestations. Apart from gastrointestinal and cardiovascular involvement, Behçet’s syndrome can involve the central nervous system (CNS) in up to 10–50 % cases [7]. The brainstem is the most common site of involvement followed by the basal ganglia and thalami [5, 7]. Brainstem lesions tend to be centred on the cerebral peduncles and the pons, and may be circular, linear, crescent-shaped or irregular in morphology. They are hyperintense on T2-weighted MR images, iso- to hypointense on T1-weighted images and exhibit variable post-contrast enhancement (Fig. 4). These may show true restriction or T2 shine through on diffusion-weighted imaging [7].

#### Vascular Malformations of the Brainstem

**Fig. 5:**
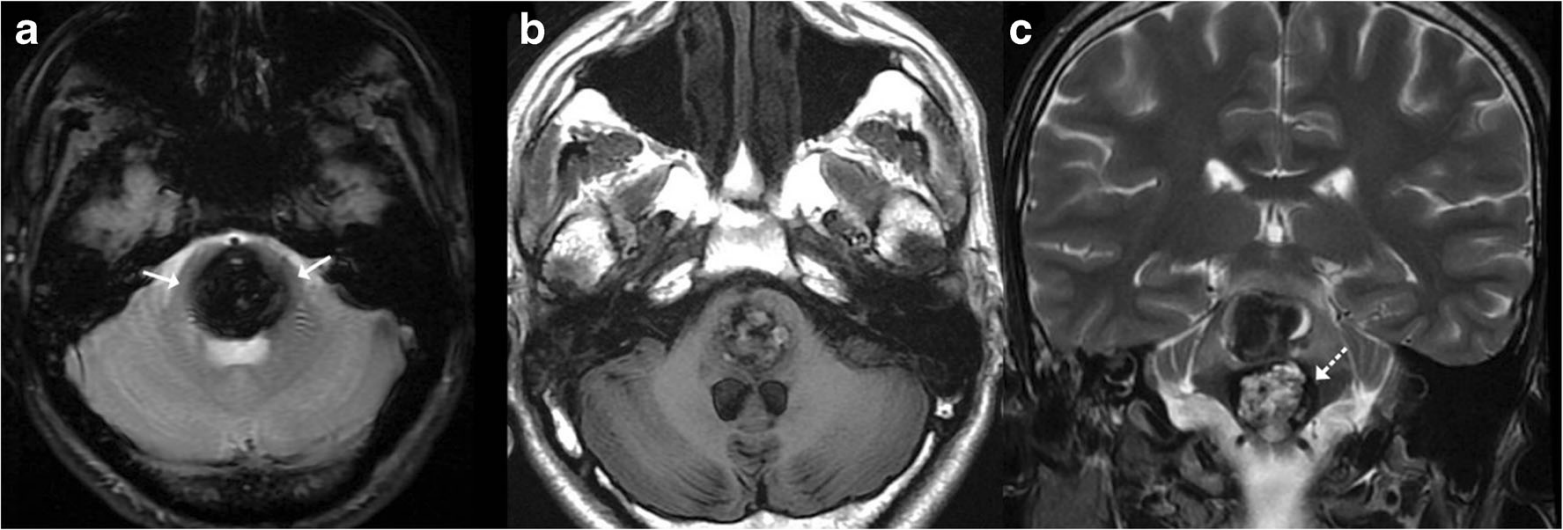
A 16-year-old patient with acute onset headache: **a** Susceptibility-weighted image shows prominent blooming in the pons (*arrow*). **b** T1-weighted images show heterogeneous areas of mixed hyper- and hypointensity consistent with blood degradation products giving a “popcorn appearance.” **c** Heterogeneous lesion with a prominent haemosiderin rim (*dotted arrow*) in keeping with cavernoma. There is an associated subacute haemorrhage in the superior aspect of the lesion

**Fig. 6:**
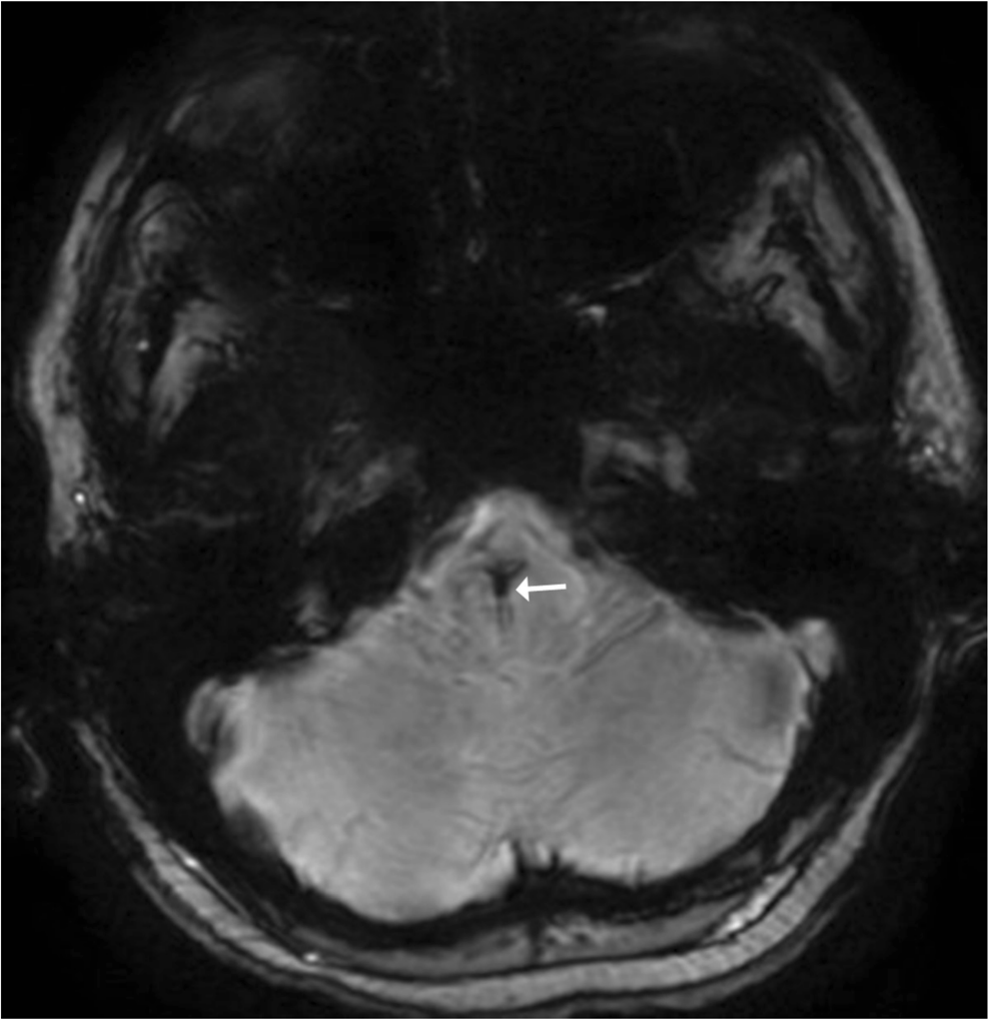
A 15-year-old patient with headache and incidental finding: Susceptibility-weighted image shows “caput medusa” appearance at the pontomedullary junction consistent with a capillary telangiectasia

Slow-flow vascular malformations include a group of angiographically occult vascular malformations which include cavernous angiomas, capillary telangiectasia, developmental venous anomalies and thrombosed arteriovenous malformations. These vascular malformations can affect the brainstem and typically remain asymptomatic, being discovered incidentally on imaging. However, they can manifest neurological symptoms, for example, because of intra-lesional bleed these may present as acute neurological deficits and altered mentation, or may even present with a more indolent course with gradually worsening neurological deficits. As elsewhere, on MRI these lesions demonstrate signal characters corroborating with various stages of blood degradation. Cavernoma or cavernous haemangiomas are a cluster of thin-walled capillaries, which typically demonstrate a characteristic “popcorn” or “berry” appearance on T1- and T2-weighted images. They classically have a rim of signal loss (haemosiderin rim) on T2-weighted images and exhibit prominent blooming on susceptibility-weighted sequences (Fig. 5). Developmental venous angiomas are a venous anomaly characterised by a “caput medusa” morphology on imaging which represents a cluster of abnormally thickened veins that drain into a single larger collecting vein, which in turn drains into either a dural sinus or a deep ependymal vein (Fig. 6). Capillary telangiectasias are vascular malformations characterised by dilated capillaries interspersed with normal cerebral parenchyma. These are typically incidentally identified and may involve the brainstem (typically the pons) and spinal cord. These are often very subtle on imaging and appear as an area of blooming on gradient sequences with no or very subtle enhancement [8, 9].

## Demyelinating Pathologies

### Acute Disseminated Encephalomyelitis

**Fig. 7:**
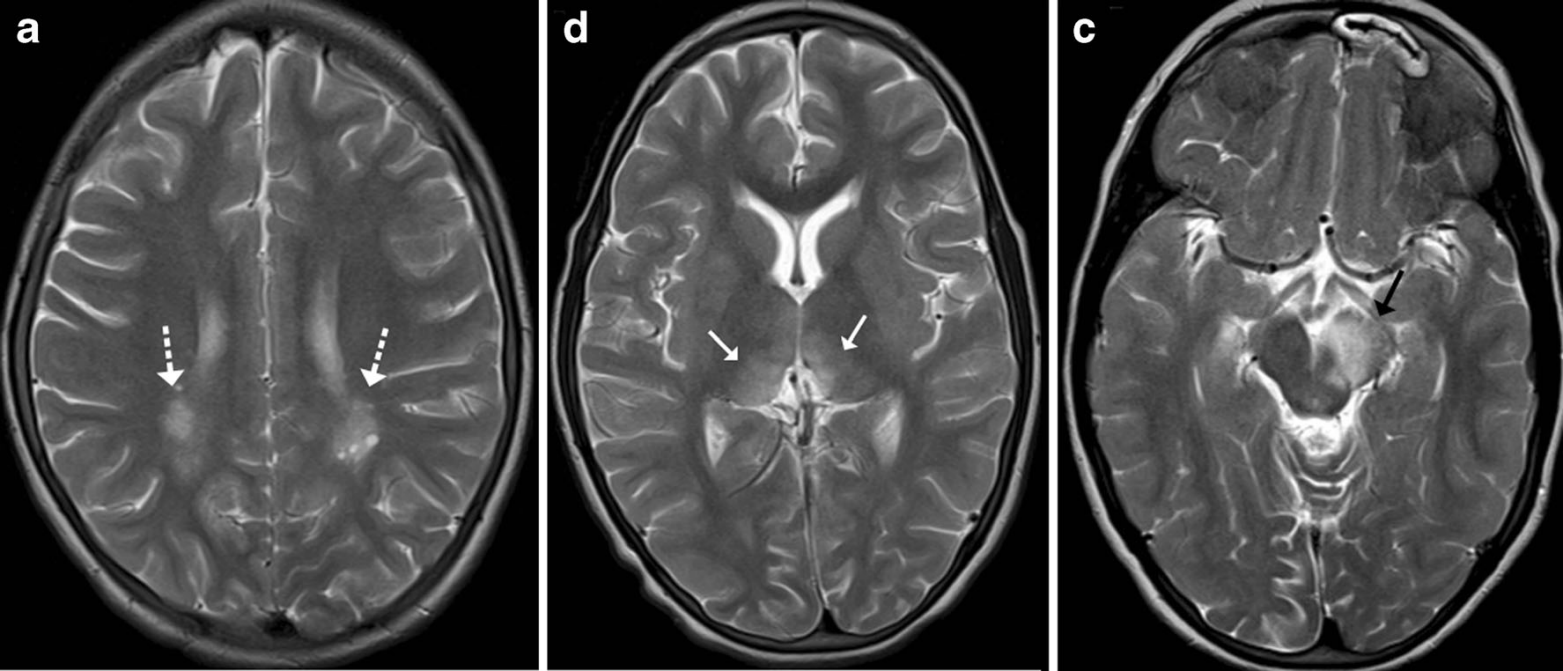
A 12-year-old boy with fever and seizures: **a**–**c** Axial T2-weighted images show hyperintense signal involving the periventricular white matter, medial thalamus and left middle cerebral peduncle suggestive of demyelination

**Fig. 8:**
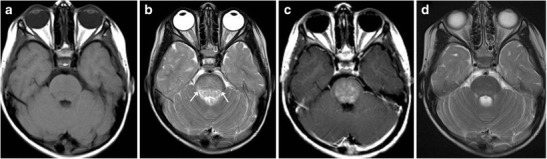
Isolated brainstem ADEM in a 12-year-old patient: **a**–**c** Axial T1-weighted image is unremarkable with hyperintense signal on T2 (*arrows*) involving the pons, which shows avid post-contrast enhancement. **d** Post treatment: Axial T2-weighted image shows near total resolution of changes in the pons

Acute disseminated encephalomyelitis (ADEM) is a monophasic, immune-mediated demyelinating CNS disorder that typically follows vaccination or viral infections. The clinical presentation is variable with no age predilection. Affected children may present with fever, headache, seizures or focal neurological deficits. MRI changes are characterised by high T2-FLAIR lesions predominantly affecting the supratentorial subcortical white matter, which tend to be asymmetric and multifocal in nature. Although the deep grey matter nuclei such as the basal ganglia and thalami may frequently become involved, brainstem involvement is quite uncommon (Fig. 7). Isolated brainstem involvement is a rare phenomenon and can be a diagnostic jigsaw both clinically and on imaging (Fig. 8). Isolated brainstem involvement has been reported as a manifestation of clinically isolated syndrome (CIS). Clinically isolated syndrome (CIS) refers to the first clinical episode of features suggestive of multiple sclerosis of which up to as many as two thirds may get converted to relapsing-remitting multiple sclerosis, although at variable speeds. Clinical response to steroids and serial MR imaging needs to be used for establishing a definite diagnosis [1, 10, 11].

### Multiple Sclerosis

**Fig. 9:**
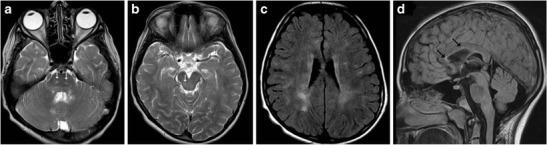
A 14-year-old patient with weakness of the limbs and dysarthria: **a**–**b** Axial T2-weighted images show ill-defined increased signal intensity in the pons and midbrain (*arrows*). **c** Axial FLAIR image shows periventricular white matter hyperintensity. **d** Sagittal FLAIR image shows increased signal in the callososeptal interface (*black arrows*) consistent with multiple sclerosis

Paediatric-onset multiple sclerosis constitutes between 2–5 % of all cases of multiple sclerosis. According to consensus definitions of the International Paediatric Multiple Sclerosis Study Group (IPMSSG), paediatric-onset multiple sclerosis may be diagnosed after two distinct episodes of CNS demyelination that are disseminated in time and space in individuals <18 years. The disease is rare before the age of 10 years and there is a female preponderance seen in children affected after the 1st decade. The imaging findings of multiple sclerosis in the paediatric age group are not significantly different from the adult-onset disease, seen as multiple sharply demarcated white matter lesions that are hyperintense on T2-weighted images exhibiting variable degree and patterns of post-contrast enhancement (Fig. 9). The incidence of tumefactive lesions and posterior fossa plaques is higher in the paediatric age group. Brainstem plaques can cause significant expansion and simulate a neoplasm [12].

### Neuromyelitis Optica (NMO)

**Fig. 10:**
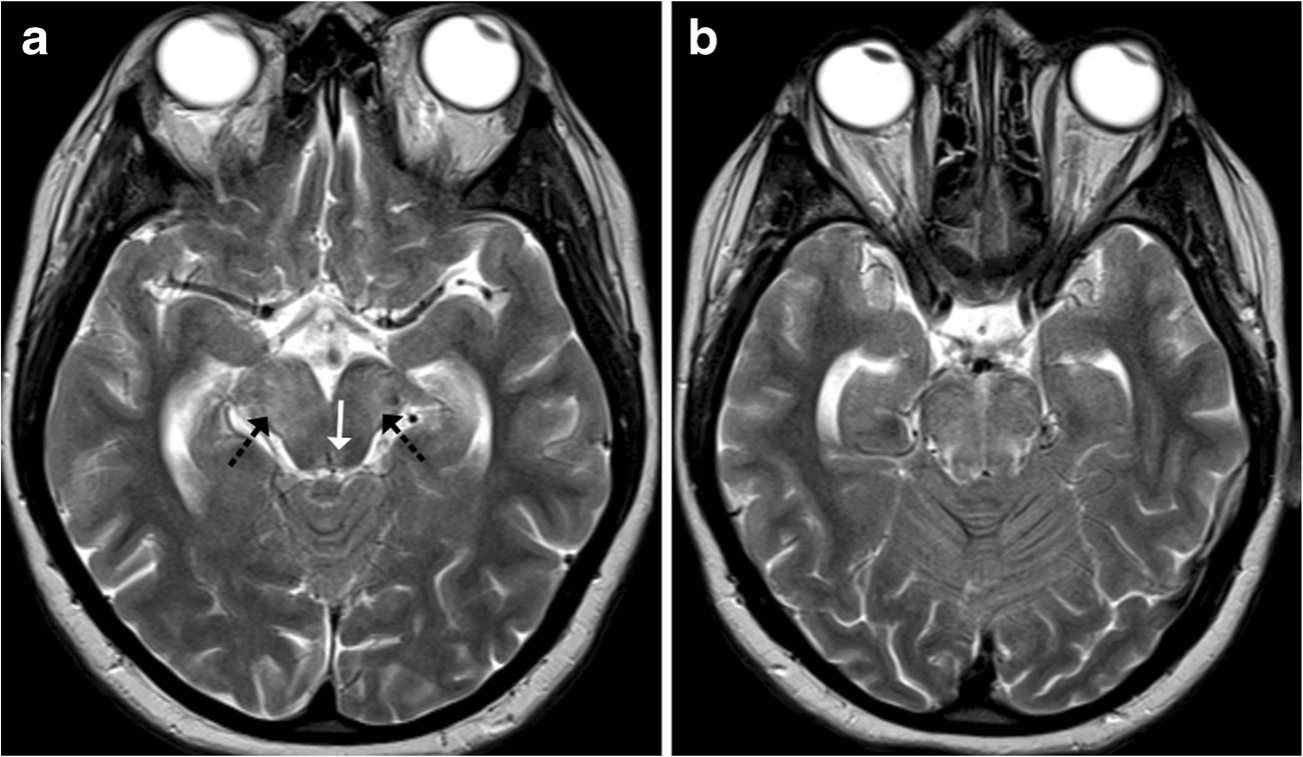
A 16-year-old female with neuromyelitis optica: **a**–**b** Axial T2-weighted images show hyperintense signal involving the tegmentum (*dotted arrow*), periaqueductal region (*arrow*) of the midbrain and pons in a diffuse pattern

Neuromyelitis optica (NMO) is a form of severe inflammatory, demyelinating disease characterised by optic neuritis and longitudinally extensive myelitis, a distinct entity from multiple sclerosis. The pathogenesis involves the formation of anti-aquaporin 4 antibodies, which may be seen in approximately 70 % of these patients. The diagnostic criteria for NMO mandates optic neuritis and myelitis to be present with either two of the following criteria, i.e., contiguous spinal cord MRI lesions extending over at least three vertebral segments, onset brain MRI not meeting the diagnostic criteria of multiple sclerosis and NMO IgG seropositivity. The most common pattern of brain lesions is non-specific foci and patches of hyperintensity in subcortical and deep white matter on T2-weighted or FLAIR images accompanied with characteristic sites of involvement including the periventricular regions of the third and fourth ventricles, midbrain and cerebellum (Fig. 10). High expression of anti-aquaporin antibodies in these locations is thought to be the credible explanation for the particular involvement of these sites [13].

### Osmotic Demyelination

**Fig. 11:**
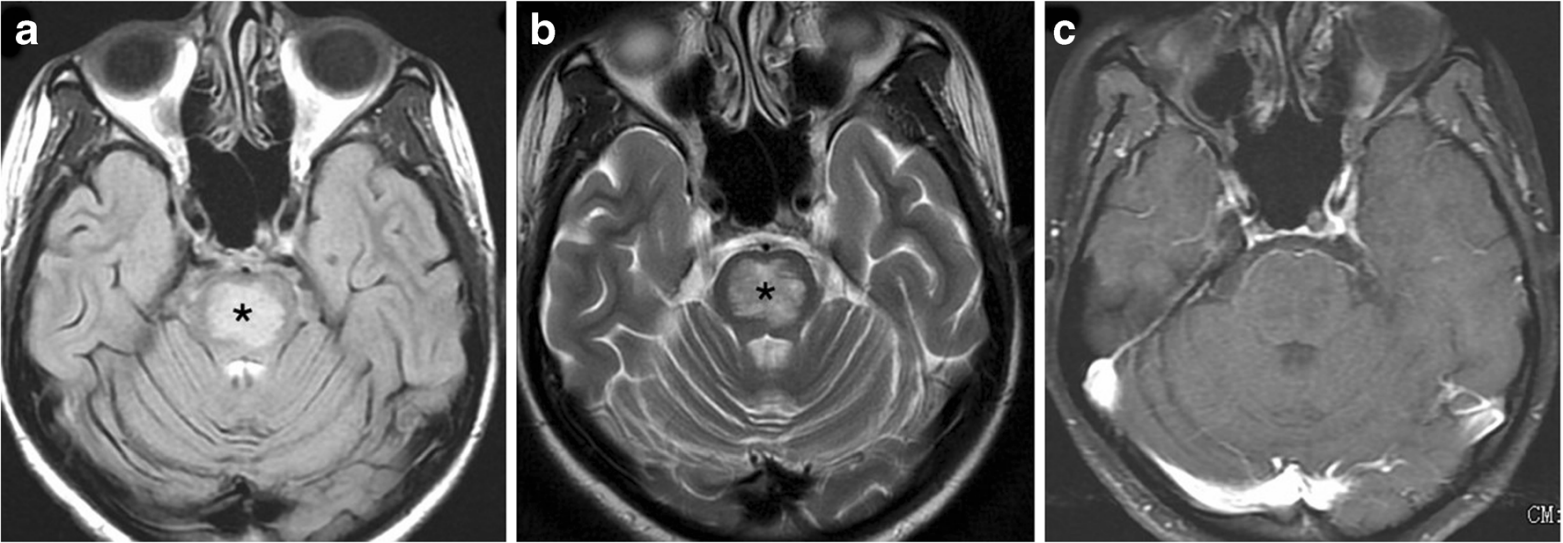
A 15-year-old female with electrolyte imbalance and altered sensorium: **a**–**b** Axial FLAIR and T2-weighted images show increased signal in central pons (*asterisk*) with characteristic sparing of the periphery. **c** No significant post-contrast enhancement is seen

Osmotic demyelination in paediatric age groups has been reported in various disorders, which include hepatic failure, liver transplantation, chronic debilitation, malnutrition and severe electrolyte disturbances. The imaging pattern in the paediatric population is similar to the adult counterparts characterised by increased signal intensity affecting the central pons on T2-weighted images. The ventrolateral pons and corticospinal tracts are characteristically spared (Fig. 11). Occasionally, there can be involvement of the extra-pontine sites, which includes the basal ganglia, thalamus and cerebellum. The treatment of the causative factors results in reversal of the imaging findings [5].

## Metabolic and Neurodegenerative Diseases

### Leigh Syndrome

**Fig. 12:**
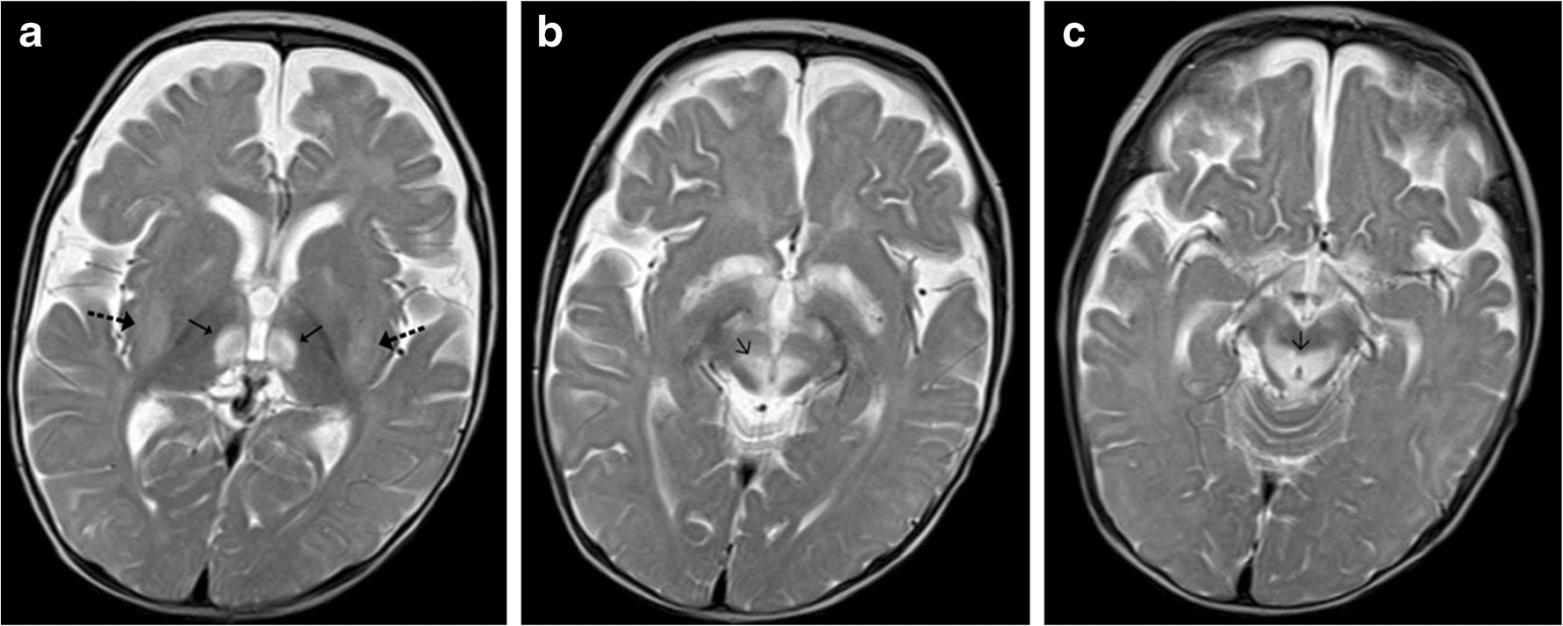
A 9-month-old child with delayed milestones and seizures: **a**–**c** Axial T2-weighted images show hyperintense signal involving the putamina (*dotted arrow*) and medial thalamic nucleus. Caudally there is involvement of the mid brain (*short arrow*) with characteristic involvement of the periaqueductal region in this patient with Leigh syndrome

Leigh syndrome, also known as subacute necrotising encephalomyelopathy (SNEM), is a form of progressive mitochondrial neurodegenerative disorder of childhood typically affecting children less than 2 years old. It is invariably fatal and clinically manifests as motor disturbances with regression of milestones, ophthalmoplegia and lower cranial nerve palsies. MR imaging reveals characteristic symmetrical T2-weighted high signal intensity in the periaqueductal white matter, medulla, midbrain and putamen (Fig. 12). Acute exacerbations may present with areas of diffusion restriction. CSF analysis reveals elevated lactate levels and MR spectroscopy may also reveal lactate elevation in the basal ganglia [5].

## Maple Syrup Urine Disease (MSUD)

**Fig. 13:**
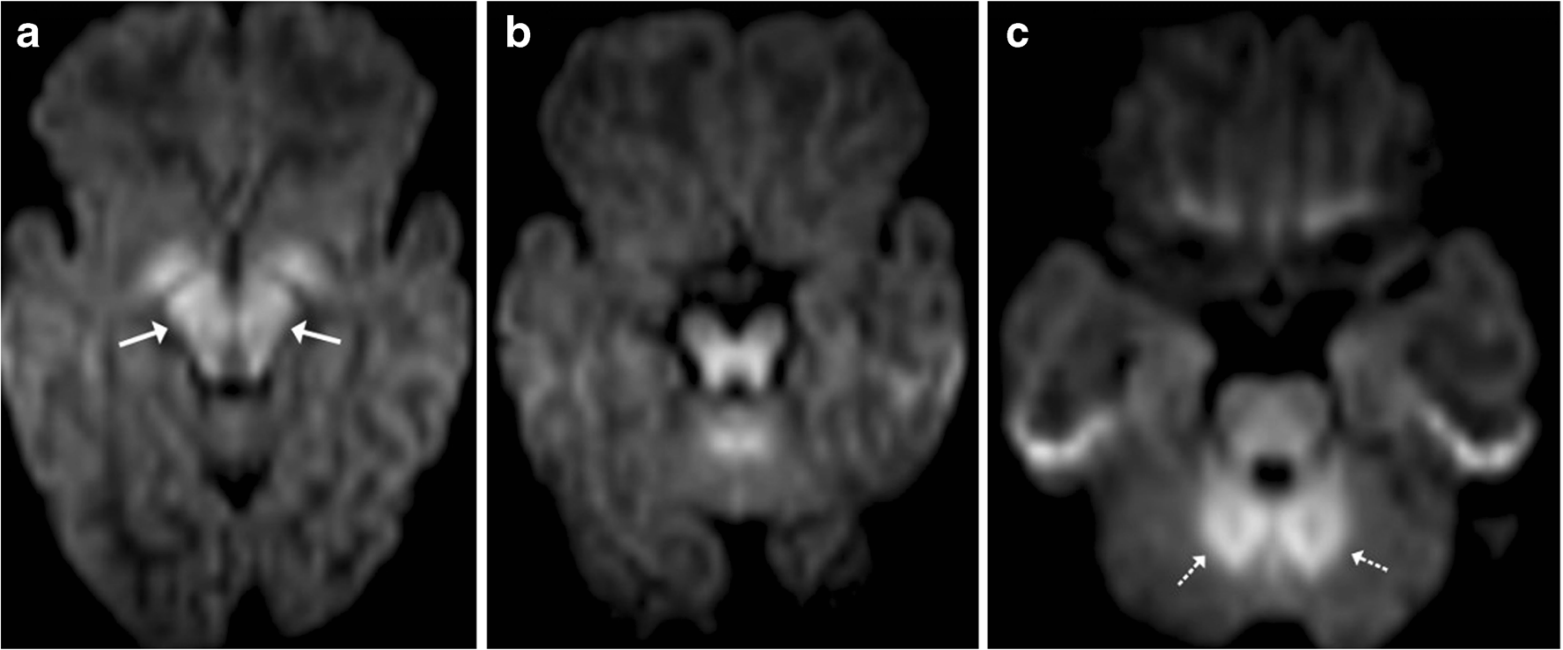
Neonate with poor feeding, lethargy and ketoacidosis: **a**–**c** Axial diffusion weighted images show restricted diffusion involving the midbrain (*arrow*), pons and cerebellar white matter (*dotted arrow*) consistent with MSUD

Maple syrup urine disease (MSUD) is an inborn error of amino acid metabolism that typically presents in the neonatal age group with a complex group of symptomatology including lethargy, poor feeding, seizures and a characteristic urine odour. Classically, MR imaging reveals diffuse brain swelling and diffusion restriction in myelinated brain areas, such as the posterior brainstem tracks and the central cerebellar white matter (Fig. 13). In the supratentorial compartment diffusion restriction may be seen along the posterior limbs of internal capsule, optic radiation and central corticospinal tracts [14].

### Wilson’s Disease

**Fig. 14:**
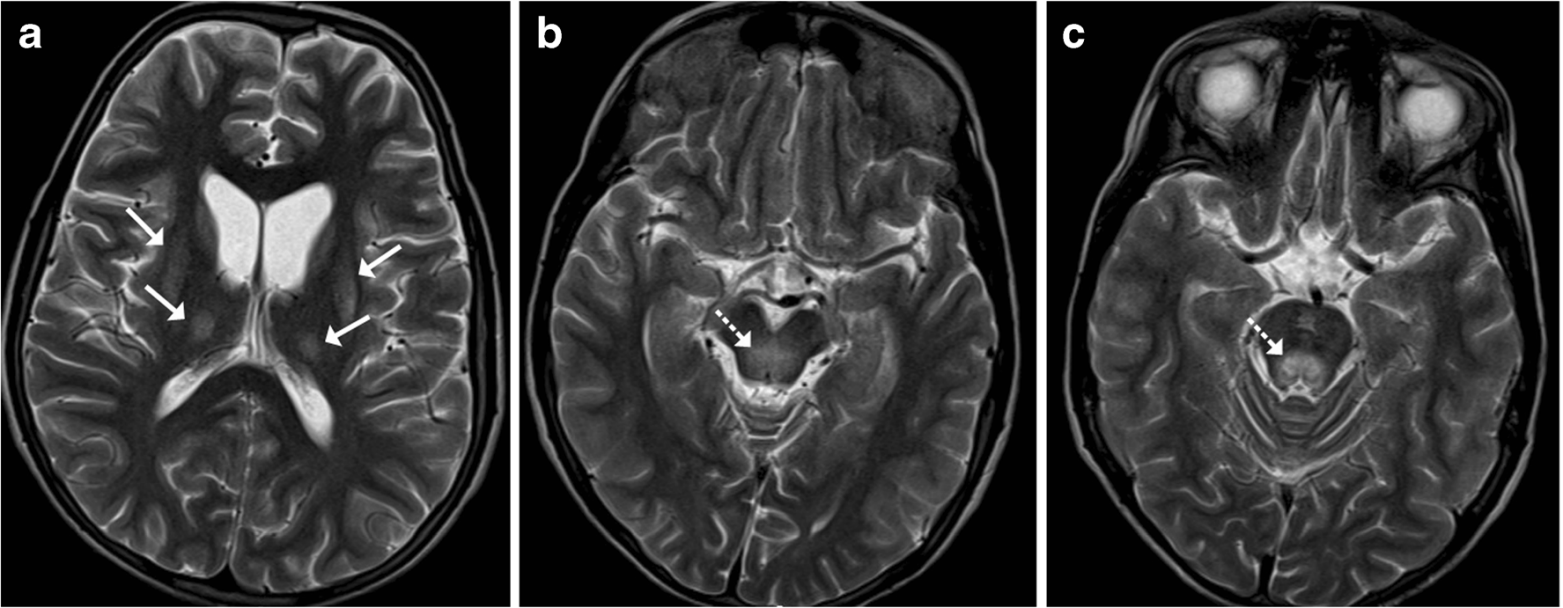
A 9-year-old patient known to have Wilson’s disease: **a** Axial T2-weighted image shows symmetrical atrophy of the basal ganglia with hyperintense signal involving the basal ganglia and thalamus (*arrows*). **b**–**c** Axial T2-weighted image shows increased signal intensity involving the midbrain as well as the pontine tegmentum (*dotted arrows*)

Wilson’s disease in an inborn autosomal recessive error of copper metabolism that affects the liver, brain and other body tissues. Children may present in the 1st or 2nd decades with features of liver disease, extra-pyramidal symptoms and Kayser-Fleischer (KF) rings observed on ophthalmoscopy. Elevated serum ceruloplasmin levels are helpful in confirming the diagnosis. Cerebral involvement typically affects the basal ganglia and thalami, which display increased T2-weighted signal intensity on MRI. The brainstem is typically involved at the midbrain and pons with T2 hyperintensity identified in these locations (Fig. 14). Occasionally, a “double panda sign” may be seen secondary to the signal changes affecting the midbrain tegmentum and pontine tectum [5, 15].

#### Haemolytic Uraemic Syndrome (HUS)

**Fig. 15:**
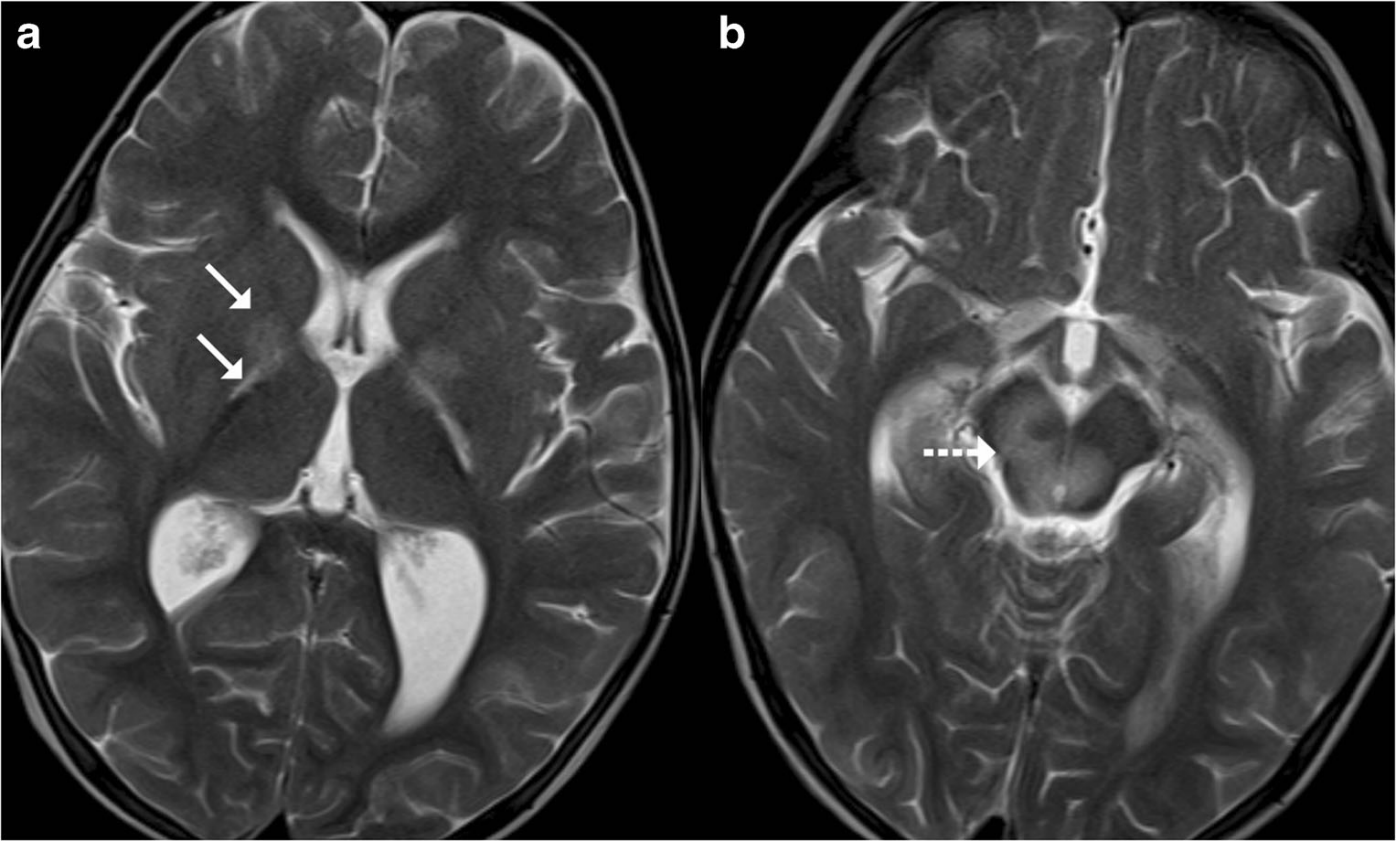
A 6-year-old patient with haemolytic uraemic syndrome and encephalopathy: **a**–**b** Axial T2-weighted images reveal increased signal in the basal ganglia, posterior limb of internal capsules (*arrow*) and midbrain (*dotted arrow*)

Haemolytic uraemic syndrome (HUS) is a complex multisystem disorder characterised by thrombocytopenia, acute renal failure and haemolytic anaemia. It is the most common cause of acute renal failure in children. CNS is the most common extra-renal site to be affected typically manifesting as altered sensorium, seizures and neurological deficits. The pathogenesis of the CNS involvement is controversial and is believed to be either toxin mediated or secondary to metabolic changes and secondary hypertension. Basal ganglia, especially dorsolateral lentiform nucleus involvement, is considered to be characteristic; arterial infarctions or white matter changes similar to posterior reversible encephalopathy can also be observed. The brainstem, cerebellum or thalami can occasionally be affected and may show foci of haemorrhage on imaging (Fig. 15) [16].

#### Posterior reversible encephalopathy syndrome

**Fig. 16:**
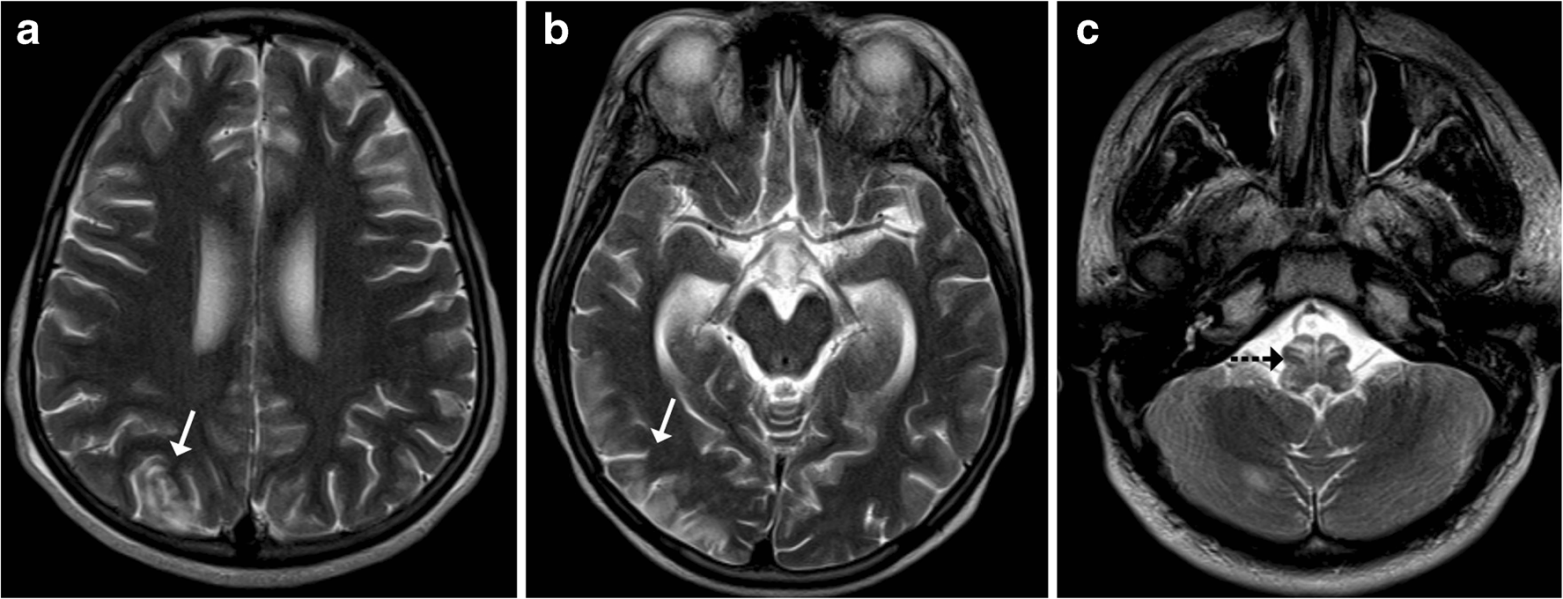
A 12-year-old patient with chronic renal failure and seizures: **a**–**c** Axial T2-weighted images reveal increased signal intensity in the right parieto-occipital cortex (*arrow*) with similar signal intensity at the cervicomedullary junction (*dotted black arrow*)

Posterior reversible encephalopathy syndrome (PRES) is a neurotoxic state that occurs secondary to failure of cerebral auto-regulation in response to acute changes in blood pressure. The disease is more commonly seen in adults; however, in the paediatric age group it can be seen in children on chemotherapy (especially in the induction phase for leukaemia), renal failure, and bone marrow or stem cell transplantation. The most common and characteristic imaging manifestation is cortical and subcortical vasogenic oedema in parieto-occipital regions. Occasionally, other parts of the cerebral hemispheres and cerebellum may be involved. The central variant is characterised by involvement of the basal ganglia and brainstem without affection of the cortical and subcortical white matter. Simultaneous involvement of the cortical-subcortical white matter and central structures may be observed sporadically (Fig. 16) [17].

#### Central tegmental tract (CTT) lesions

**Fig. 17:**
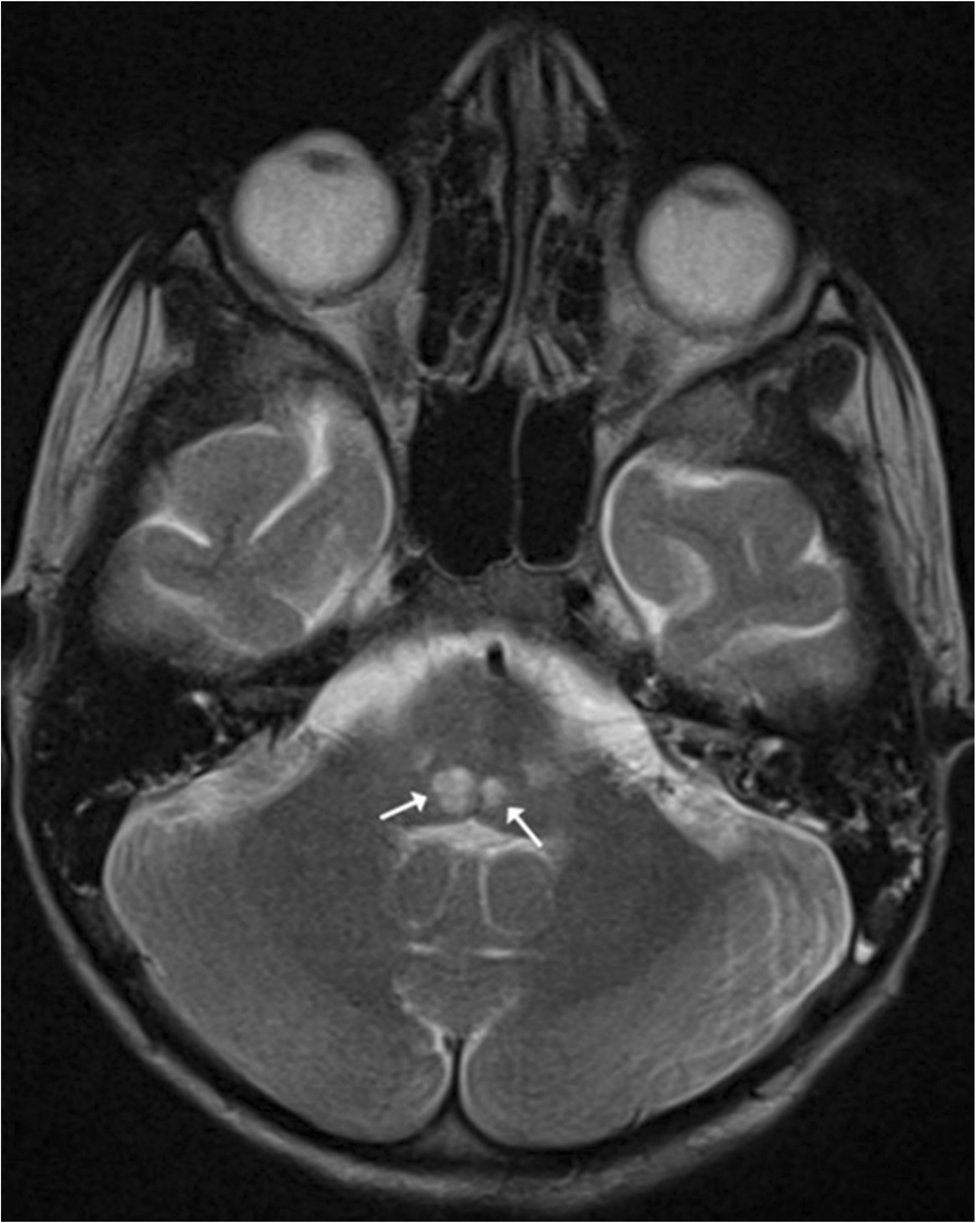
A 3-year-old patient with cerebral palsy and delayed milestones: Axial T2-weighted image reveals focal tegmental hyperintensities at the level of medulla (*arrows*)

**Fig. 18:**
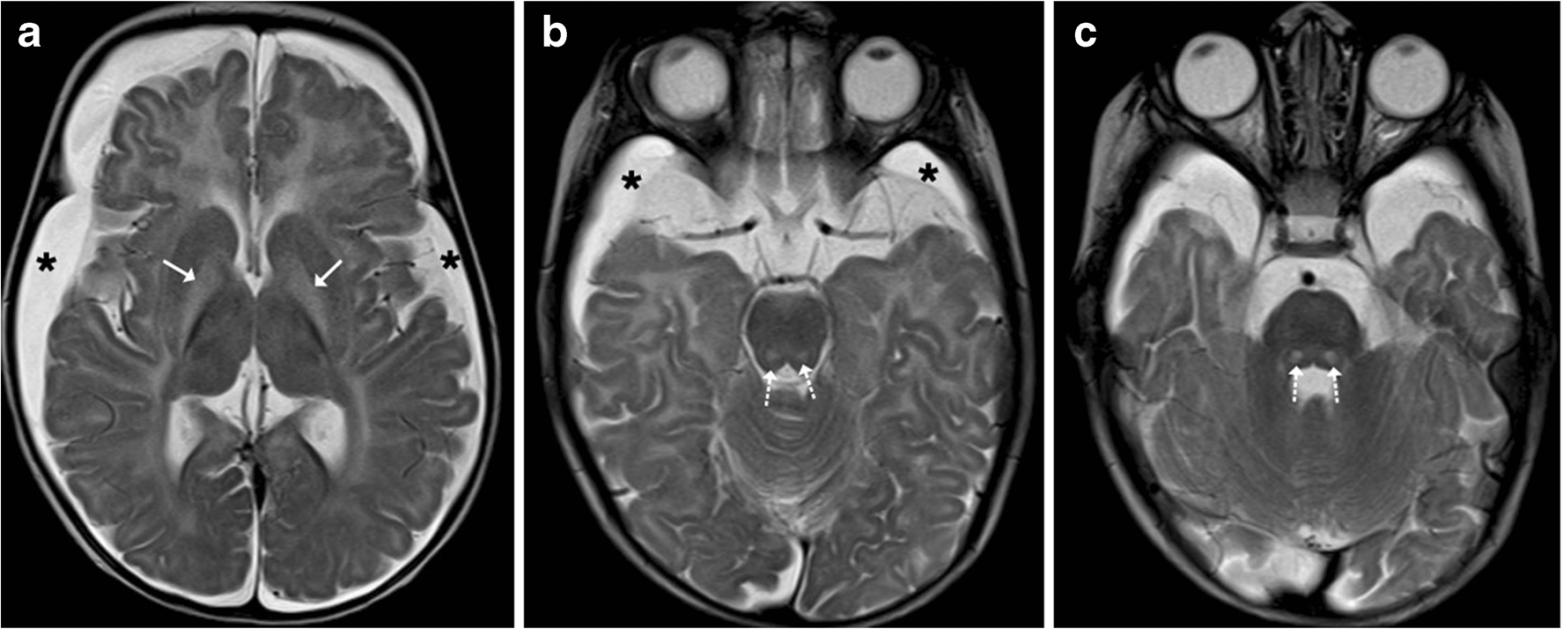
An 8-month-old child with glutaric aciduria presenting with altered sensorium: Axial T2-weighted image shows hyperintensity involving the globus pallidi (*arrow*) and bilateral subdural hygromas (*asterisk*). Prominence of the CSF spaces is noted anterior to the temporal lobe. Increased signal intensity is noted in the pontine tegmentum (*dotted arrow*)

The central tegmental tract (CTT) is primarily the extrapyramidal tract connecting the red nucleus and inferior olivary nucleus. CTT lesions refer to symmetrical foci of T2 hyperintensity that involve the tegmentum and can be encountered in a variety of clinical conditions. Whilst cerebral palsy (Fig. 17) is the most common of them, CTT lesions have also been documented in congenital metabolic disorders such as non-ketotic hyperglycenemia, MSUD, mitochondrial encephalopathy, glutaric aciduria type 1 (Fig. 18), hypoxic injury and drug toxicity [18].

### Brainstem encephalitis

The causes of brainstem encephalitis can be variable, ranging from infections to autoimmune aetiology. Common infectious agents responsible for brainstem encephalitis include listeria, tuberculosis, viruses, fungi and parasites.

## Viral Encephalitis

**Fig. 19:**
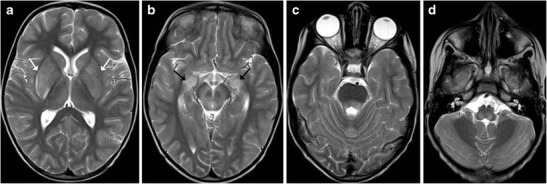
A 5-year-old patient with a history of dog bite and comatose state during imaging: **a**–**d** Axial T2-weighted images reveal increased signal intensity in the basal ganglia (*white arrow*) with increased signal intensity involving the medial thalami, hippocampi (*black arrow*), midbrain, pons and medulla

**Fig. 20:**
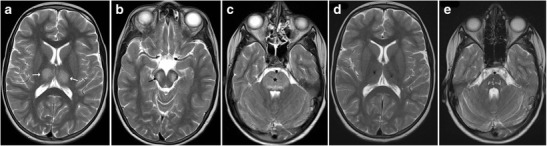
A 10-year-old patient with fever and altered sensorium: **a**–**c** Axial T2-weighted images show near symmetrical increased signal intensity in the medial thalami (*white arrow*), middle cerebral peduncles (*black arrow*) and pons (*asterisk*). The possibility of viral encephalitis, likely Japanese encephalitis, was considered. **d**–**e** Follow-up MRI: Axial T2-weighted images show hypointense foci (possibly remote haemorrhage) in the thalami with volume loss and an area of gliosis in the pons

Most cases of viral encephalitis have a very similar clinical and imaging manifestations and differentiation solely based on imaging alone can be extremely challenging. Rabies encephalitis has a propensity to affect the spinal cord, brainstem, basal ganglia, thalami, hypothalamus and hippocampi (Fig. 19); needless to say, that the clinical information is paramount as a very similar involvement can be seen in other causes of viral encephalitis. Similarly, in a clinical context of suspected viral encephalitis, involvement of the brainstem with bilateral thalamic involvement can suggest Japanese encephalitis (Fig. 20). Herpes encephalitis apart from the limbic system can also involve the brainstem [19, 20].

## Tuberculosis and Fungal

**Fig. 21:**
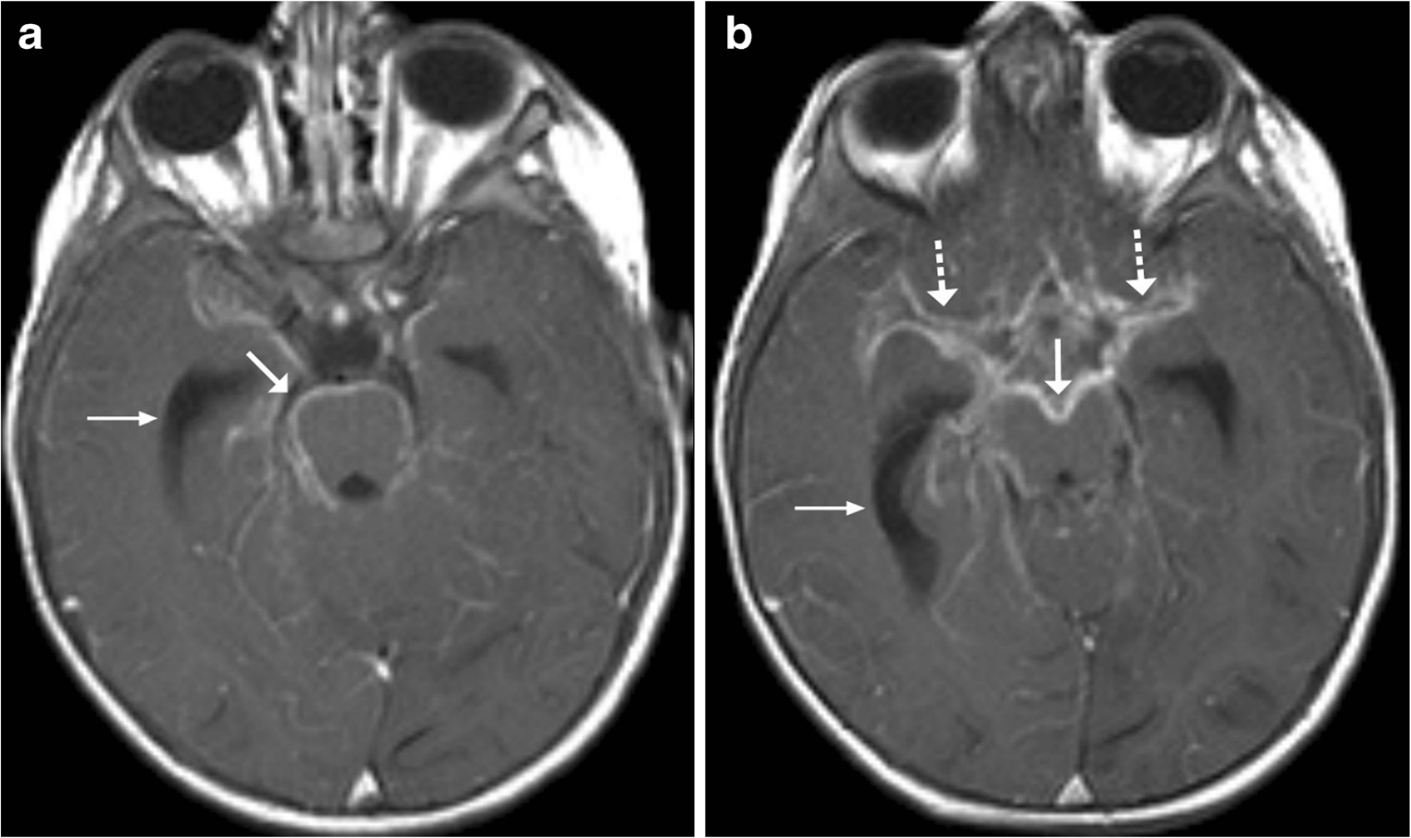
A 6-year-old patient with fever and headache: **a**–**b** Axial contrast enhanced T1 image shows marked meningeal enhancement in basal cisterns (*arrow*), middle cerebral artery cisterns (*dotted arrows*) and dilated lateral ventricles (*thin arrows arrow*) in a case of tubercular meningitis

**Fig. 22:**
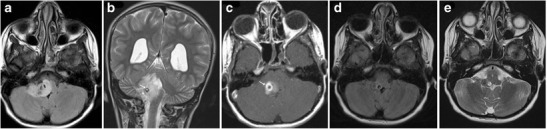
6 year old with headache, vomiting and fever: **a** Axial FLAIR image reveals hyperintense signal involving the medulla with extension into the adjacent cerebellar white matter. **b** and **c** Coronal T2-weighted image reveals small lesion with peripheral hypointensity at pontomedullary junction (*arrow*), the lesion shows peripheral enhancement on post contrast images. Possibility of CNS tuberculoma was considered. **d**–**e** Follow-up after 3 months on anti-tubercular treatment: Axial FLAIR and T2-weighted images show significant reduction in the hyperintensity involving the medulla with residual lesions. Clinically the child showed significant improvement

**Fig. 23:**
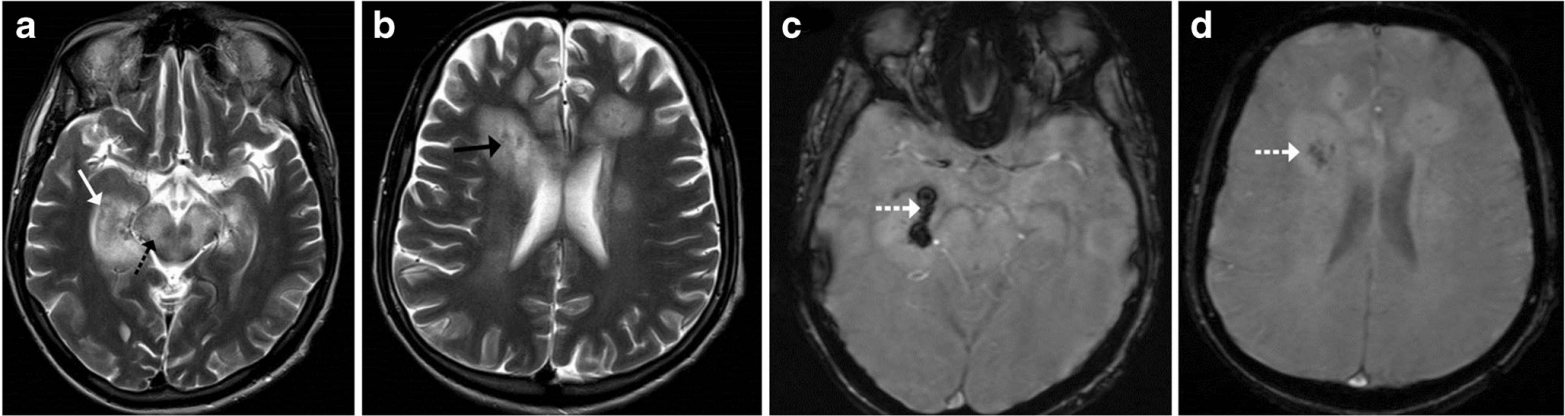
A 16-year-old patient with acute promyelocytic leukaemia on chemotherapy with angioinvasive aspergillosis: **a**–**b** Axial T2- weighted MRI shows increased signal intensity in the temporal lobes (*white arrow*) with involvement of the cerebral peduncles. Similar signal intensity is noted in the frontal white matter bilaterally. **c**–**d** Significant blooming (*dotted arrows*) is noted on susceptibility-weighted imaging

Brainstem involvement by tuberculosis can be isolated or as a part of disseminated CNS disease. Leptomeningeal involvement can be in the form of basal meningitis (Fig. 21). The signal intensity of the parenchymal lesions vary on T1-and T2- weighted sequences depending on the stage of the granuloma though most of these lesions tend to show peripheral rim enhancement following intravenous contrast administration (Fig. 22) [21, 22]. Understandably, isolated brainstem lesions can be extremely challenging as the appearances may be indistinguishable from neoplastic processes like gliomas. Fungal infections of CNS are usually opportunistic in nature occurring in immunocompromised patients thus clinical history can prompt the radiologist to consider them in appropriate settings. Intracranial involvement is either secondary to haematogenous dissemination or extension from the adjacent sinus disease. The frequent occurrence of significant parenchymal destruction is often associated with high fatality. Mucormycosis and aspergillosis owing to their angioinvasive propensity tend to result in in parenchymal haemorrhages which can be helpful in clinching the diagnosis (Fig. 23) [22].

## Parasitic (Cerebral Malaria)

**Fig. 24:**
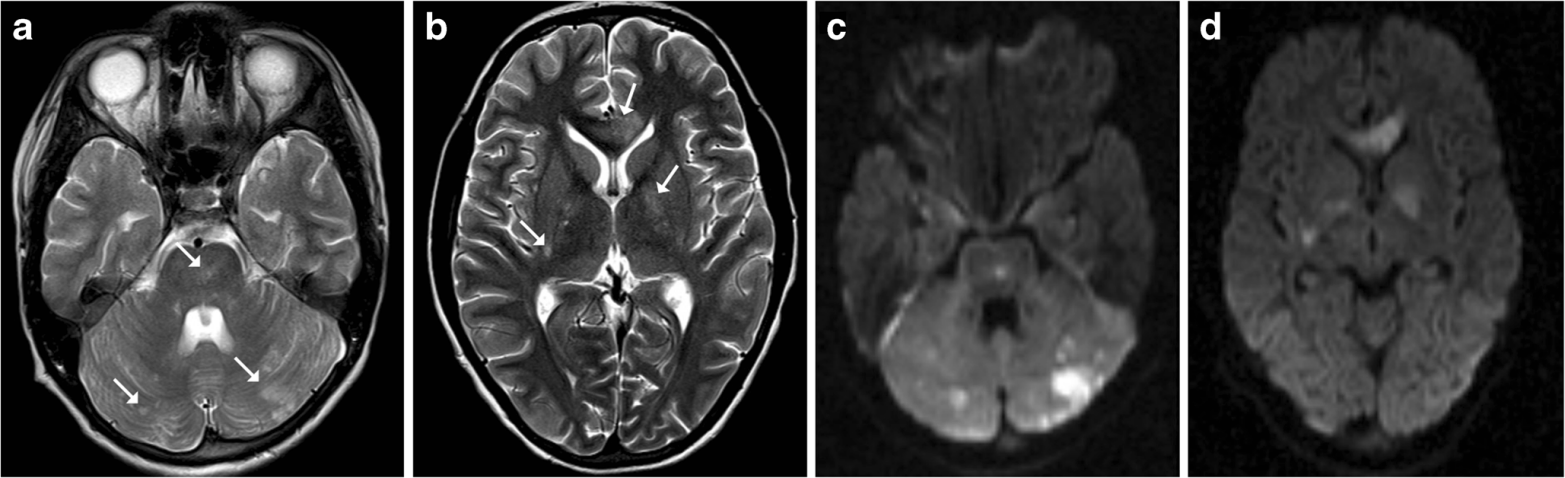
A 13-year-old patient with cerebral malaria: Axial T2-weighted MRI reveals hyperintense foci showing diffusion restriction in the left cerebellum, pons, bilateral basal ganglia and corpus callosum (*arrows*) corresponding to areas of cytotoxic oedema

Cerebral manifestations in malaria occur because of sequestration of infected erythrocytes in the microcirculation resulting in multiple foci of ischaemic necrosis. It typically manifests as encephalopathy and coma and is frequently fatal. MRI features are highly variable and may include multiple cortical infarcts with or without involvement of the deep grey matter nuclei, corpus callosum, cerebellum and brainstem. Involved areas exhibit T2-FLAIR hyperintensity and the presence of multifocal areas of diffusion restriction connote cytotoxic oedema. Frequently, there are foci of haemorrhages that are best appreciated on gradient sequences (Fig. 24) [22, 23].

## Tumours of the Brainstem

### Brainstem Glioma

**Fig. 25:**
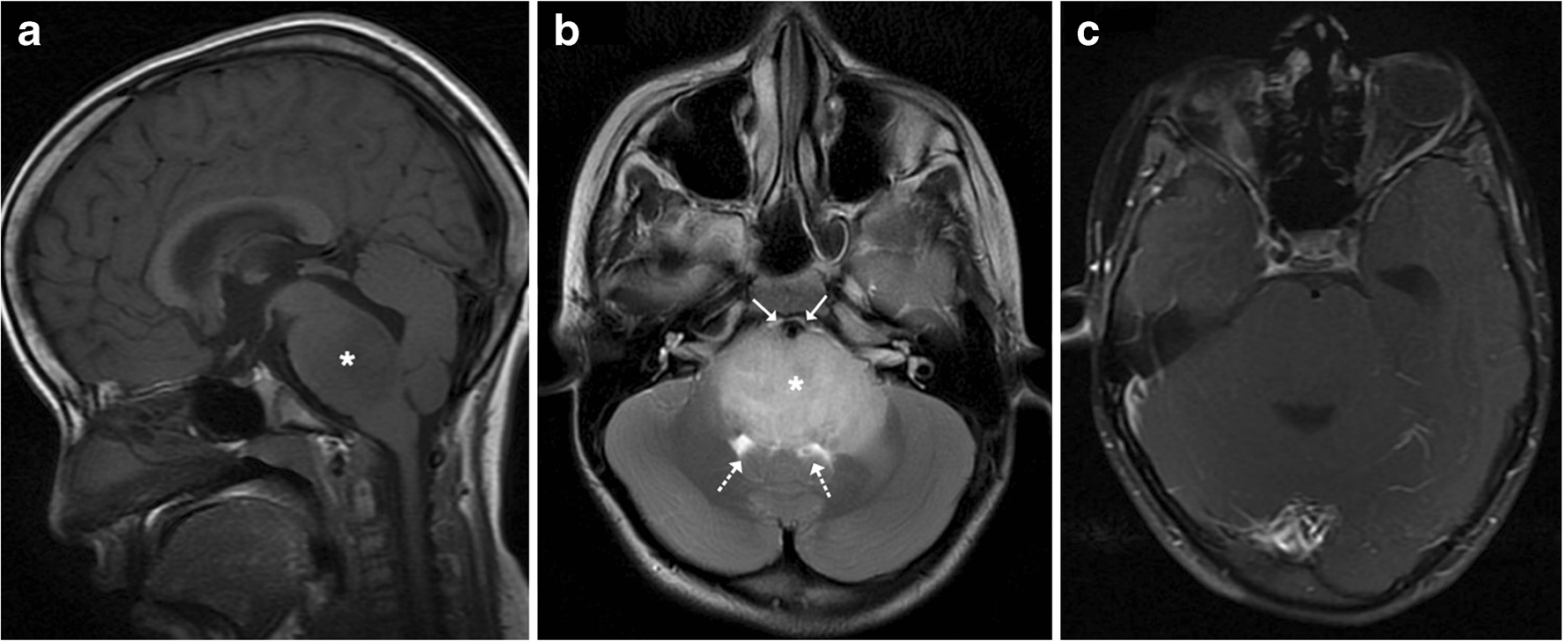
A 9-year-old child with headache and vomiting: **a** Sagittal T1-weighted image shows expansion of the pons with hypointense signal (*asterisk*). **b** Axial T2-weighted image shows increased signal intensity involving the pons (*asterisk*) with the basilar artery (*arrow*) appearing engulfed by the lesion. Posteriorly the fourth ventricle is compressed (*dotted arrow*) (flat fourth ventricle sign). **c** No significant contrast enhancement is noted within the lesion

**Fig. 26:**
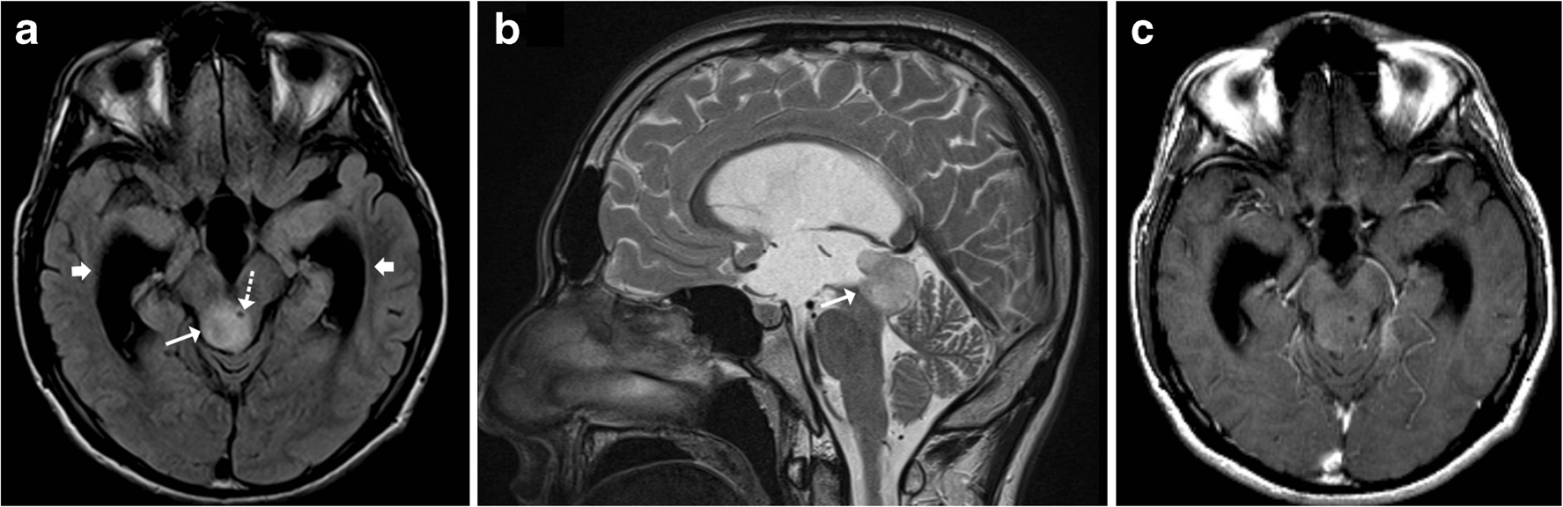
A 16-year-old male with headache and vomiting: **a**–**b** Axial FLAIR and T2-weighted sagittal MRI shows a relatively well-defined hyperintense lesion involving the tectum (*arrow*). The aqueduct is encased and compressed (*dotted arrow*) with resultant dilatation of the temporal horns (*block arrow*). **c** No significant contrast enhancement is noted in keeping with a glioma

**Fig. 27:**
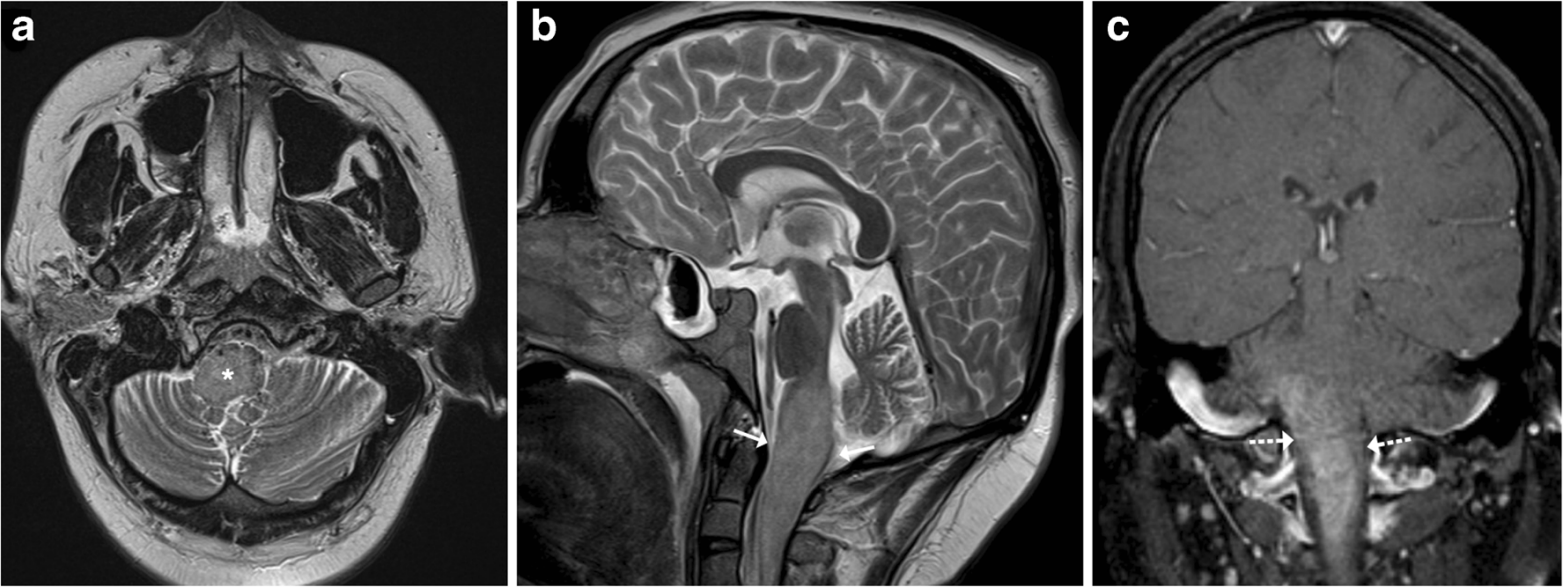
A 15-year-old male with altered headache and lower cranial nerve palsies: **a**–**b** Axial and sagittal T2-weighted image reveals an ill-defined hyperintense expansive lesion involving the medulla (*), which is extending caudally to involve the cervical cord (*arrow*). **c** Post contrast the lesion shows homogeneous enhancement (*dotted arrows*)

Brainstem gliomas comprise a heterogeneous group of tumours having variable histological patterns and prognostic implications. These lesions comprise 10–20 % of the intracranial tumours in the paediatric age group with a majority of them occurring in the first decade of life (peak incidence: 3–7 years). The lesions are sub-classified as diffuse, focal, exophytic and cervicomedullary. Diffuse lesions tend to affect the pons and present as long tract signs; clinically, ataxia or lower cranial nerve palsies are associated with a dismal prognosis. These lesions expand the pons and cause anterior displacement of the basilar artery, which appears engulfed by the lesion. There can be flattening of the fourth ventricle resulting in hydrocephalus (*flat fourth ventricle sign*). Most lesions are hypo- to isointense on T1-weighted images, hyperintense on T2-weighted images, and display no or minimal enhancement (Fig. 25). Tectal gliomas tend to be focal and have a better prognosis as these are low-grade astrocytomas. These lesions typically cause mild expansion of the tectal plate resulting in narrowing of the aqueduct and obstructive hydrocephalus. The lesions are isointense on T1-weighted, hyperintense on T2-weighted images and do not show post-contrast enhancement (Fig. 26). Medullary tumours have a variable morphology and may be exophytic, focal or diffuse in nature. Imaging findings are non-specific although mostly associated with the expansion of the medulla. Diffuse lesions can extend cranially to involve the pons and caudally to involve the cervical spinal cord (Fig. 27). The enhancement characteristics of these lesions are inconsistent and variable [24].

**Fig. 28:**
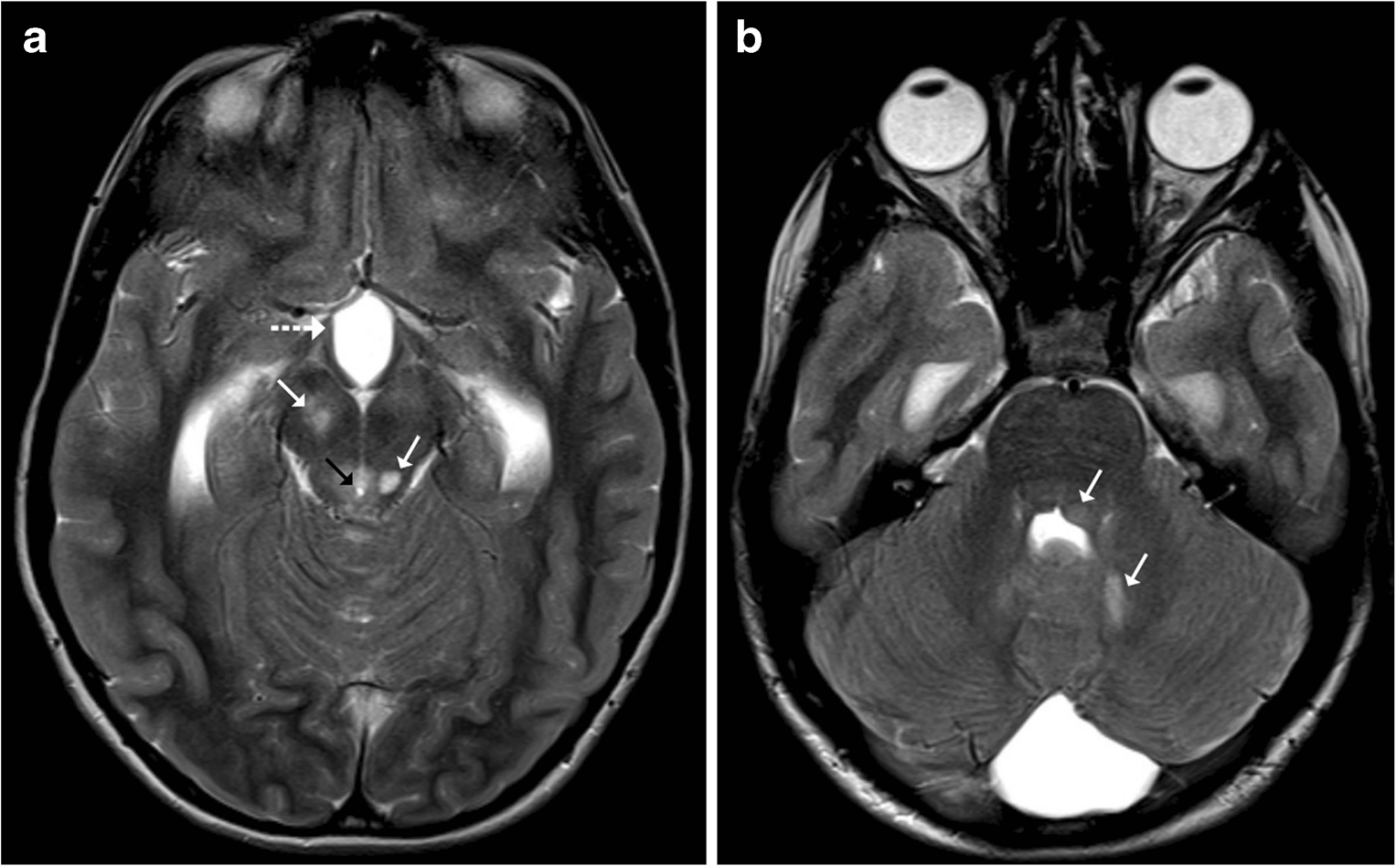
A known case of neurofibromatosis type I with hydrocephalus: **a**–**b** Axial T2-weighted images show mild expansion of the midbrain and pons with multiple hyperintense lesions involving the midbrain, pons and cerebellar peduncle (*arrow*). Periaqueductal hyperintensity with narrowing of the aqueduct (*black arrow*) resulting in third ventricular dilatation (*dotted arrow*)

Pseudotumoral focal or diffuse enlargement of the brainstem (without neoplastic involvement) can be encountered in neurofibromatosis type I. There may be associated T2 hyperintense foci or hamartoma; these have a predilection for the brainstem, cerebellar peduncles and basal ganglia. Occasionally, patients may develop hydrocephalus secondary to a tectal hamartoma or a glioma causing secondary aqueductal stenosis (Fig. 28) [25, 26].

#### Secondary involvement of paediatric brainstem

**Fig. 29:**
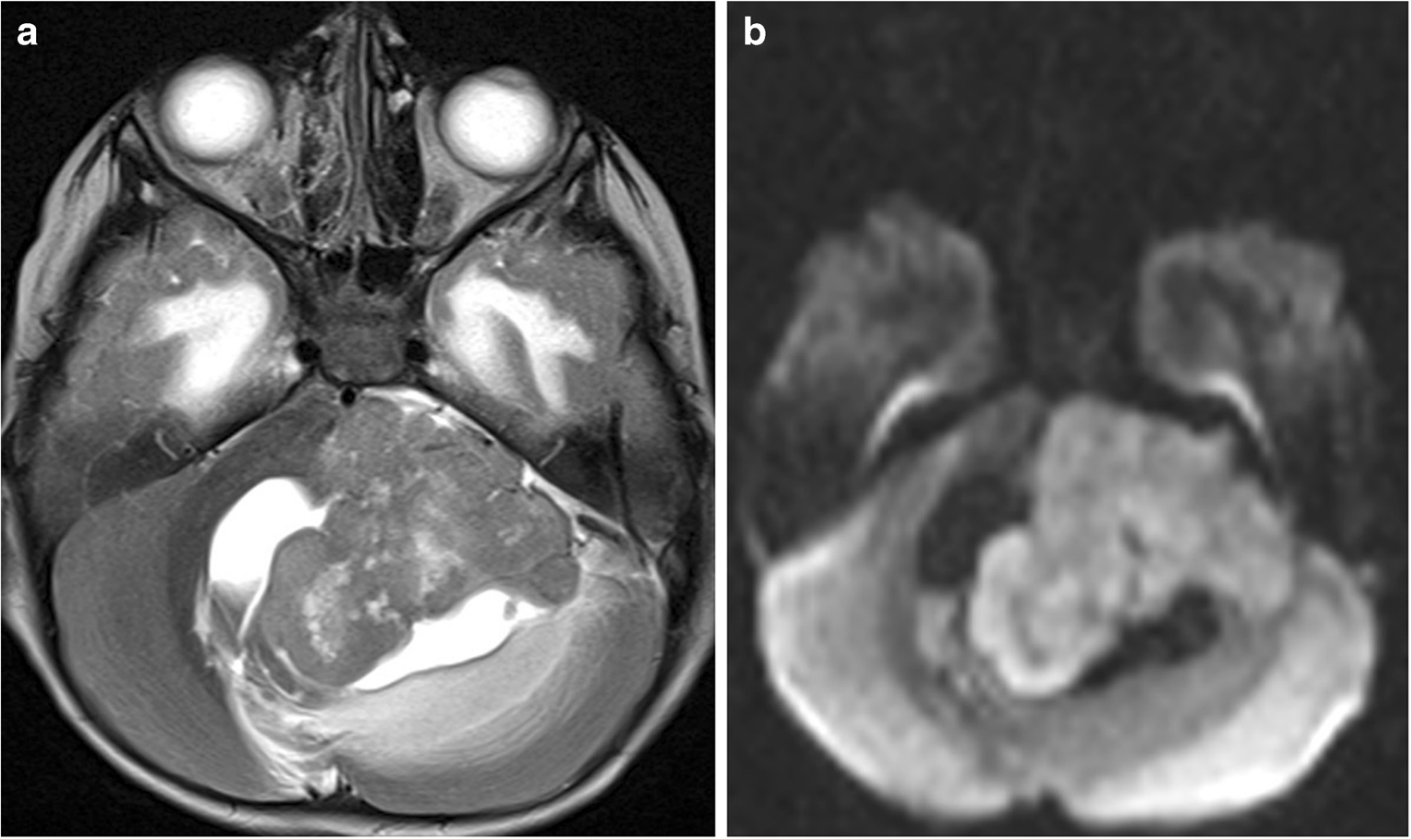
A 5-year-old patient with cerebellar medulloblastoma: **a**–**b** Axial T2-weighted and diffusion-weighted image shows a large hypointense mass showing restriction in the left cerebellar hemisphere causing severe brainstem compression

It is crucial to remember that the brainstem can become secondarily involved because of direct extension of other CNS tumours especially from the posterior fossa region. Brainstem compression and/or infiltration by tumours such as medulloblastoma or gliomas is also not uncommon (Fig. 29) [24].

**Fig. 30:**
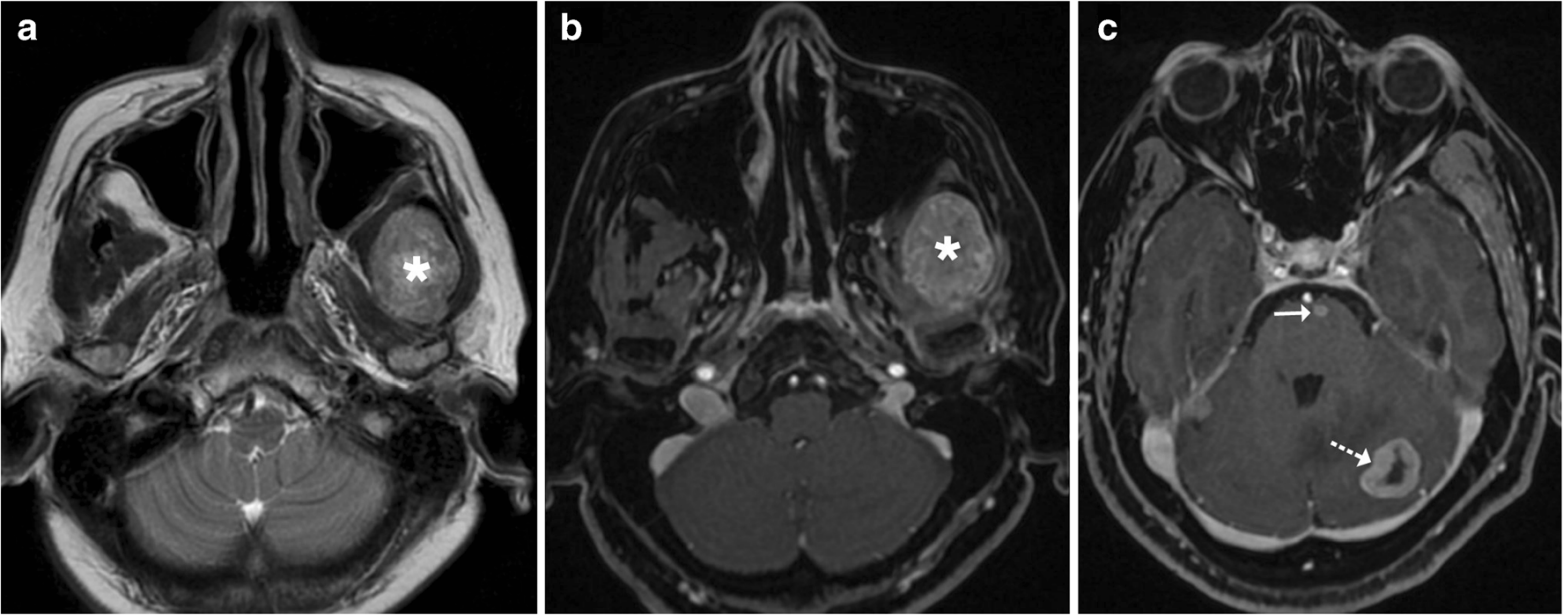
A 17-year-old patient with acute myeloid leukaemia: **a**–**b** Axial T2 and post-contrast T1-weighted image shows an enhancing mass in the left masticator space suggestive of a chloroma (*asterisk*). **c** Axial T1-weighted image shows enhancing deposits in the left cerebellum (*dotted arrow*) and pons (*arrow*)

Extra-cranial solid tumour metastasising to the brain is a rarity in the paediatric age group though the brain can be a frequent site of involvement in haematological malignancies such as leukaemias. Brain involvement in leukaemia can be direct (due to leukaemic infiltration) (Fig. 30) or indirect. Indirect involvement occurs secondary to thrombosis or haemorrhage (due to hyperleucocytosis, sepsis and coagulopathy), or secondary to a neutropenic state predisposing the patient to infections (see Fig. 23), or as a sequela of chemotherapy-related encephalopathy or thrombotic microangiopathy [27].

## Conclusion

A multifarious paediatric neurological disorders may primarily or secondarily involve the brainstem. Whilst these can be aetiologically classified as infections, metabolic and demyelinating disorders, vascular conditions, neurodegeneration and tumours, they often have overlapping clinical presentation. Moreover, their deep-seated nature and high risk of potential morbidity often precludes histopathological assessment. Thus their evaluation as well as follow-up primarily revolve around multi-planar MR imaging, which not only helps in localising the pathology but also aids in formulating a rational diagnosis. Whilst certain disorders such as Leigh syndrome and Maple syrup urine disease have certain characteristic imaging patterns, it is vital to understand that neither imaging nor clinical assessment alone may be able to clinch the diagnosis. Thus we as radiologists ought to be aware of the salient clinical features, laboratory parameters and radiological features that need to be looked into before we venture into reporting these pathologies. We hope that the comprehensive diagnostic approach (Table 2) outlined in this article will not only help the radiologists in acquainting themselves with the spectrum of paediatric brainstem pathologies, but also motivate them to work in close collaboration with their clinical counterparts, which would ensure improved clinical care offered to their paediatric patients.

**Table 2:** Salient clinical and imaging features of brainstem lesions in the paediatric age group

Aetiology	Clinical features	Imaging features
Vascular		
Infarction	Acute neurological deficit Bulbar symptoms to comatose state Associated conditions: cardiac disease, haemolytic anaemia or chemotherapy related	T2/FLAIR hyperintensity + diffusion restriction
Hypoxic ischaemic encephalopathy	Birth asphyxia Obstructed labour Poor Apgar score	Typical sites of involvement: ventrolateral thalamus, posterior limb of the internal capsule and peri-Rolandic cortex. Dorsal brainstem involvement suggests profound hypoxia
Vascular malformations	Asymptomatic Acute neurological deficit in acute bleed Progressive neurological deficits	Cavernous angioma: “popcorn” appearance Developmental venous anomaly: “caput medusae” appearance
Vasculitis (Behçet’s disease)	Peak in 2nd decade Multisystem disorder involving the oral cavity, genitalia and eyes Skin, joints and other major systems may also be involved	T2 hyperintense lesions with variable post-contrast enhancement Sites: brainstem > basal ganglia > thalamus
Demyelination		
ADEM	Recent vaccination or viral illness Monophasic disease	Asymmetric T2-hyperintense subcortical white matter lesion + basal ganglia and thalami involvement Brainstem involvement: uncommon. Isolated brainstem involvement: extremely rare
Multiple sclerosis	More common in 2nd decade with female preponderance Progressive disease with chronic relapsing and remitting course	Posterior fossa and brainstem involvement frequent in paediatric age group
Neuromyelitis optica	Optic neuritis Clinical features of long segment myelitis	T2 hyperintense lesions characteristically in periventricular location around the third, fourth ventricles and midbrain and cerebellum Increased signal intensity in the optic nerves, chiasma as well as the spinal cord
Osmotic demyelination	Electrolyte disturbances Rapid correction of sodium Malnutrition Transplantation	T2 hyperintense signal in the central pons with characteristic sparing of the ventrolateral pons and cerebrospinal tracts Basal ganglia and thalami may be involved
Metabolic, toxic and neurodegenerative diseases		
Leigh syndrome	Typical age group <2 years Hypotonia Neurological regression Elevated serum or CSF lactate levels	T2 hyperintense signal in basal ganglia, periaqueductal white matter, medulla and midbrain Diffusion restriction in acute states Lactate peak in affected regions
Maple syrup urine disease	Neonatal onset Lethargy Seizures Characteristic urine odour	Diffuse brain swelling Diffusion restriction in posterior brainstem tracks and the central cerebellar white matter
Wilson’s disease	Signs of hepatic involvement KF ring Extrapyramidal signs Raised serum ceruloplasmin	Basal ganglia and thalami typically involved in symmetrical pattern Midbrain and pons involvement can be seen Double panda sign
Haemolytic uremic syndrome	History of diarrhoeal infection followed by acute renal failure, thrombocytopenia and haemolytic anaemia	Basal ganglia especially dorsolateral lentiform nuclei involvement + arterial infarctions + patterns of reversible encephalopathy
Posterior reversible encephalopathy syndrome	Elevated blood pressure Children with renal failure or on chemotherapeutic agents	Typically involves parieto-occipital cortex or subcortical white matter. Atypical cases may involve the brainstem and basal ganglia
Central tegmental tract lesions	Non-specific finding seen in various conditions such as cerebral palsy, metabolic disorders and drug toxicity	Symmetrical T2-hyperintense foci involving the midbrain, pontine or medullary tegmentum
Brainstem encephalitis		
Viral encephalitis	Fever, altered sensorium Bulbar symptoms History of canine bite in suspected rabies encephalitis	Herpes encephalitis: bilateral/unilateral temporal lobe ± brainstem involvement Japanese encephalitis: bilateral thalamic with brainstem involvement Rabies: cord, brainstem, basal ganglia, hippocampal and hypothalamic involvement
Tuberculosis	Hydrocephalus CSF analysis Immunocompromised patients	Basal meningitis, variable signal intensity of granuloma, ring enhancement
Fungal	Immunocompromised patients Sinus disease	Haemorrhages, infarctions secondary to angioinvasive nature
Cerebral malaria	Fever Altered sensorium Endemic regions	Multiple acute infarcts affecting various parts of the brain
Tumours		
Brainstem glioma	Most common in 1st decade, peak between 3–7 years Signs of raised intracranial tension Long tract signs with cranial nerve palsies	Pontine glioma: non- enhancing expansive lesion, typically engulfs basilar artery, flat fourth ventricle sign Tectal glioma: non-enhancing lesions ± obstructive hydrocephalus Medullary glioma: focal, exophytic or diffuse lesion. Variable enhancement pattern
Neurofibromatosis I	Family history Cutaneous stigmata such as café au lait spots, neurofibromas, etc.	Non-neoplastic brainstem enlargement T2 hyperintense lesions—hamartoma or glioma Aqueductal stenosis and hydrocephalus
